# Mangiferin mitigates dexamethasone-induced insulin resistance in rats: insight into vascular dysfunction and hepatic steatosis

**DOI:** 10.3389/fphar.2025.1572758

**Published:** 2025-05-08

**Authors:** Abdullah Q. Alsaedi, Manar A. Nader, Dalia H. El-Kashef, Marwa E. Abdelmageed

**Affiliations:** ^1^ Department of Pharmacology and Toxicology, Faculty of Pharmacy, Mansoura University, Mansoura, Egypt; ^2^ Department of Quality and Output Control, Branch of Ministry of Health, Madinah, Saudi Arabia; ^3^ Department of Pharmacology and Toxicology, Faculty of Pharmacy, Mansoura National University, Gamasa, Egypt

**Keywords:** mangiferin, dexamethasone, VCAM, IRS1/AKT, NLRP3/NF-κB/TNF-α, insulin resistance/*Wistar* rats

## Abstract

**Aim:**

Insulin resistance (IR) is a hazard to human health in which peripheral insulin-target organs, like the liver, become less sensitive to normal levels of insulin. Dexamethasone (DEX)-induced IR is a distinct model of IR. Hence, the present study investigates the efficacy of mangiferin (Mang) in the reversal of DEX-induced IR in the livers and aortas of rats.

**Main methods:**

Rats were randomly assigned into six groups: control (CTRL), Mang, DEX, and three pretreated groups (received Mang 25 mg/kg, 50 mg/kg, or 100 mg/kg, orally for 14 days, with DEX (1 mg/kg) injected from day 8 to day 14). On day 15, serum, liver, and aorta tissues were obtained and examined using biochemical, histological, and immunohistochemical assessments.

**Key findings:**

Mang administration attenuated DEX-induced IR, evidenced by decreased oral glucose tolerance test (OGTT) and fasting serum insulin levels, in addition to improving the DEX-induced hepatic and aortic histopathological alterations. Additionally, Mang attenuated DEX-induced alterations in liver function parameters and improved serum lipid profiles, oxidative stress, and antioxidant biomarkers. Mang also markedly increased hepatic and aortic levels of insulin receptor substrate 1 (IRS1), protein kinase B (AKT), AMP-activated protein kinase (AMPK), and peroxisome proliferator-activated receptor-gamma (PPAR-γ) levels. Mang reduced hepatic and aortic tumor necrosis factor-alpha (TNF-α), forkhead box protein O1 (FOXO-1), hepatic NOD-like receptor family pyrin domain-containing 3 (NLRP3), phosphoenol pyruvate carboxy kinase (PEPCK), and glucose 6-phosphatase (G6Pase). Mang elevated hepatic glycogen synthase kinase3 (GSK3α) and glycogen synthase (GS2) levels. Furthermore, Mang ameliorated aortic expression levels of endothelin-1 (ET-1), vascular cell adhesion molecule-1 (VCAM), c-Jun N-terminal kinase (JNK), nuclear factor kappa B (NF-κB), and vascular endothelial growth factor (VEGF) and increased endothelial nitric oxide synthase (eNOS) and prostacyclin (PGI2) levels.

**Conclusion:**

Mang administration could confer hepato- and vasculo-protective activity via its hypolipidemic, hepatoprotective, anti-inflammatory, and antioxidant efficacy.

## Highlights

• Mang attenuated DEX-induced IR by decreased OGTT and fasting serum insulin levels.
• Mang improved DEX-induced elevated serum levels of hepatic function biomarkers.
• Mang improved serum lipid profile, oxidative stress, and antioxidant biomarkers.
• Mang increased hepatic and aortic levels of IRS1 and AKT.
• Mang lowered hepatic and aortic TNF-α and hepatic NLRP3 levels.
• Mang ameliorated aortic ET-1, VCAM, JNK, and NF-κB and increased eNOS levels.

## 1 Introduction

Insulin resistance (IR) is a physiological state in which cells are unable to respond to the standard activities of the hormone insulin (Seong et al., 2019; Liu et al., 2024). High blood glucose from the body results in insulin production, but the cells become resistant to it and are unable to use it as efficiently (Stanford and Goodyear, 2014). As a result of IR, the liver releases glucose into the blood as a consequence of increased glycogenolysis and gluconeogenesis, leading to diminished utilization of blood glucose, ultimately causing hyperglycemia (Huang et al., 2018).

Phosphatidylinositol 3-kinase (PI3K)/protein kinase B (AKT) signaling pathways are the key paths of insulin signal transduction, and insulin receptor substrate (IRS) is deemed a crucial molecule in PI3K/AKT signaling pathway. Insulin receptor substrate1 (IRS1) is one of the subunits and is strictly associated with insulin-linked carbohydrate metabolism. Next, insulin interacts with the insulin receptor, and the IRS1 is motivated, which recruits the downstream signal transduction path (Zhang et al., 2019; Li et al., 2020), as phosphorylated IRS1 interacts with PI3K, and phosphoinositide-dependent kinase-1 (PDK-1) promotes the phosphorylation of AKT resulting in AKT activation, which is a significant influence in the insulin-signaling pathway (Feng et al., 2021). AKT is a significant molecule downstream of PI3K, where it subsequently triggers AKT and performs a necessary function in organizing glucose metabolism by stimulating glycogen synthesis and inhibiting gluconeogenesis. It also contributes to numerous signaling pathways and is necessary for glycogen synthesis, glucose transport, and apoptosis (Lee et al., 2020; Taheri et al., 2024). In addition, AMP-activated protein kinase (AMPK) plays an important role in promoting glucose uptake in an insulin-independent manner (Kahn, 1996). Studies have reported that enhancing AMPK signaling and phosphorylation elevates glucose transporter 4 (GLUT4) expression, which augments glucose uptake in various cell types. As AMPK is an important cellular regulator of glucose metabolism, it has been considered a potential therapeutic target to ameliorate IR in treating T2D (Shamshoum et al., 2021). Forkhead box protein O1 FOXO1 transcription factors contribute to different tissues, including the liver, heart, and vessels. Phosphorylated (inactivated) FOXO1 proteins are triggered by oxidative stress and contribute to ROS-induced cell damage and apoptosis (Ponugoti et al., 2012). Evidence shows that activation of FOXO transcription factors reduces the level of oxidative stress by generating anti-oxidative enzymes that break down ROS, such as catalase and manganese superoxide dismutase, which enhance catalase activity and downregulate oxidative stress (Salih and Brunet, 2008). Peroxisome proliferator-activated receptor-gamma (PPAR-γ) can facilitate fatty acid metabolism and reduce the levels of circulating lipids (Koh et al., 2003). PPAR-γ activation reduces hyperglycemia by increasing sensitivity to peripheral insulin and decreasing the production of hepatic glucose (McGuire and Inzucchi, 2008). PPAR-γ is reported to activate PI3K/AKT signaling by activating p38 mitogen-activated protein kinase (MAPK) and focal adhesion kinase sensors, which have been demonstrated to regulate IR and sensitivity (Lee et al., 2016).

It has been demonstrated extensively that an AKT-mediated inactivation of glycogen synthase kinase (GSK)-3 contributes to a reduction in the net phosphorylation and, subsequently, activation of glycogen synthase (GS) (Kvandová et al., 2016). Regarding gluconeogenesis, the ability of insulin to suppress PEPCK transcription is sensitive to PI3-kinase inhibition (Mukohda et al., 2017; Tanaka et al., 2017).

Insulin-resistant individuals are at 2.5 times higher risk of dying of cardiovascular problems than non-insulin-resistant people (Kewalramani et al., 2008). The problems occur due to dyslipidemia, endothelial dysfunction, and sympathetic overactivity (DeFronzo and Ferrannini, 1991). The endothelium, a specialized and extensive tissue in the body, regulates the normal function of blood vessels by acting as a mechanical lining. It also plays a pivotal role in regulating leukocyte adhesion, platelet aggravation, and blood vessel patency and controls the release of secretory factors in response to mechanical stimuli (Cahill and Redmond, 2016). The major role of the endothelium is to ensure adequate blood flow, which depends on the counterbalance between vasodilators and vasoconstrictors. Vasodilators, including prostacyclin (PGI2) and nitric oxide (NO), aim to maintain adequate blood by dilating the vessels, while vasoconstrictors, including endothelin-1 (ET1) and thromboxane A2 (TXA2), counterbalance the excessive vasodilation and maintain the vascular tone (Godo and Shimokawa, 2017).

Early studies revealed that inflammation affected IR (Kern et al., 2003; Odegaard and Chawla, 2013). It has been shown that inflammation is an important factor leading to IR, and inflammatory components, such as interleukins, tumor necrosis factor (TNF-α), and nuclear factor kappa B (NF-κB), could affect insulin sensitivity and islet beta cell performance via blood or paracrine, which in turn causes IR (Wei et al., 2018).

The aorta, as the body’s largest artery, plays a crucial role in vascular function and is sensitive to metabolic changes. IR can lead to endothelial dysfunction, affecting vascular tone and blood pressure regulation. By examining the aorta in rat models, researchers can assess how IR impacts vascular health. The effects of insulin and free fatty acids have been investigated on matrix metalloproteinases in aortic tissue, providing insights into vascular changes associated with IR (Coutinho and Chapman, 2011).

Dexamethasone (DEX) is a fluorinated steroid and a synthetic glucocorticoid that exhibits significantly higher potency in its glucocorticoid activity than the naturally occurring cortisol hormone (Dubashynskaya et al., 2021). When DEX is given in excess to humans or experimental animals, it causes IR, and many studies have used it to induce IR in rodents (Severino et al., 2002). The core pathways of DEX-induced IR are multifaceted and comprise various organs, such as the liver, where DEX stimulates gluconeogenesis and glycogenolysis, directing an increase in hepatic glucose output (Kuo et al., 2015). The adverse effects of DEX may be reversible if given for the short term; however, patients receiving DEX for the long term may suffer from irreversible metabolic damage (Rafacho et al., 2010), as well as the production of free radicals such as hydrogen peroxide, superoxide, and hydroxyl radicals, which contribute to oxidative stress and deteriorates insulin secretion and action, hastening the onset of type 2 diabetes mellitus (T2D) (Bjelaković et al., 2007). Glucocorticoid administration causes IR, leading to various cardiovascular complications (Soccio et al., 2014). Glucocorticoids induce changes in endothelium and also decrease aortic compliance, which may be secondary to IR (Kumar et al., 2015).

Mangiferin (Mang; 1,3,6,7-tetrahydroxyxanthone-C2-β-D-glucoside) is a naturally occurring C-glucosyl xanthone in plants such as *Mangifera indica, Mangifera persiciformis*, and *Anemarrhena asphodeloides* (Niu et al., 2012; Benard and Chi, 2015). Mang has been reported to have many beneficial biological actions such as antidiabetic, anti-inflammatory, and antioxidant actions (Miura et al., 2001b; Saha et al., 2016). It also has antiproliferative activity by inducing apoptosis by suppressing the activity of the molecule NF-κB (Shoji et al., 2011). Recent investigations revealed that Mang meaningfully decreased the level of plasma triglycerides in diabetic rats (Miura et al., 2001b; Muruganandan et al., 2005). Mang effectively prevents hepatic steatosis by encouraging inhibition of the proteins involved in the *de novo* lipogenesis pathway in the liver (Guo et al., 2011; Xing et al., 2014).

IR is still recognized as an enigma. Despite the number of widely available therapeutic approaches, the demand for precise, safe, and effective therapy is increasing as treatment of IR is still challenging for scientists, medical doctors, and all the people affected, directly or circumstantially, by this disorder. In view of the complex pathogenesis of IR, natural drugs have become new players in diabetes and IR prevention and treatment because of their wide targets and few side effects. To the best of our knowledge, the effect of Mang on hepatic and vascular dysfunction induced by DEX in rats had not been reported before. Because there is almost no data on the effects of Mang on IR induced by glucocorticoids, the rationale of our study was designed to assess the protective effect of Mang on DEX-induced IR in rats and pinpoint the mechanistic pathways underlying such effects on this model involving mitochondrial stress leading to IR by focusing on two target organs: the liver and the aorta.

## 2 Materials and methods

### 2.1 Animals

Thirty-six male *Wistar* rats 3 months old, weighing 180–250 g, were obtained from the Egyptian Organization for Biological Products and Vaccines (VACSERA), Giza, Egypt. During the study, rats were kept at constant environmental and nutritional conditions throughout the experimental period, and potential environmental stressors that may lead to stress and distress, including levels of ambient light, noise, vibrations, fluctuations, or extremes in temperature, were avoided to minimize animal suffering. Room temperature ranged between 20°C and 26°C with regular light cycles of 12/12–10/14 h light/dark, relative humidity at the level of rat cages of 40%–70% with room ventilation rates of approximately 15–20 air changes/hr. Rats were left to acclimatize for 1 week prior to the conduction of the experimental protocol. All animals had unrestricted access to food and water. Animals were monitored at least once daily. All procedures were carried out following the ethical strategies permitted by the Animal Care and Use Committee, Mansoura University, Egypt, as a part of project code number MU-ACUC (PHARM.PHD.23.01.12), and the animal study was conducted in compliance with ARRIVE guidelines.

### 2.2 Drugs and chemicals

DEX was obtained as DEX sodium phosphate ampoule (EIPICO, Egypt). Mang was purchased in the form of powder from Sigma-Aldrich, MO, United States and was provided orally as a suspension in 0.5% carboxy-methyl cellulose (CMC). All other chemicals and reagents were of universal chemical value.

### 2.3 Study design

Rats (total number 36) were allocated to six groups, each consisting of six rats, at the same time using simple (or unrestricted) randomization, which excluded all other variables as follows:

1. Group I (CTRL group, n = 6): performed as normal control, received 0.5% CMC by oral gavage for 14 days, and were injected with normal saline (0.9% NaCl) (5 mL/kg) for the last 7 successive days.
2. Group II (Mang CTRL group, n = 6): received Mang (100 mg/kg, orally) (Niu et al., 2012; Zhang et al., 2023) once daily by oral gavage for 14 days and were injected with 0.9% NaCl (5 mL/kg, intraperitoneally (i.p.)) for the last 7 successive days.
3. Group III (DEX group, n = 6): received 0.5% CMC by oral gavage for 14 days and were injected with DEX (1 mg/kg, i.p.) (Mokuda et al., 1991; Protzek et al., 2016; El-Sonbaty et al., 2019; Poualeu Kamani et al., 2022) for the last 7 consecutive days.
4. Group IV (Mang 25 + DEX, n = 6): received Mang (25 mg/kg, orally) (Modupe and Oladiji, 2016) once daily by oral gavage for 14 days and were injected with DEX (1 mg/kg) for the last 7 sequential days.
5. Group V (Mang 50 + DEX, n = 6): received Mang (50 mg/kg, orally) (Guo et al., 2011; Zhang et al., 2023) once daily by oral gavage for 14 days and were injected with DEX (1 mg/kg, i.p.) for the last 7 continual days.
6. Group VI (Mang 100 + DEX, n = 6): received Mang (100 mg/kg, orally) (Niu et al., 2012; Zhang et al., 2023) once daily by oral gavage for 14 days and were injected with DEX (1 mg/kg, i.p.) for the last 7 succeeding days.

### 2.4 Body weight change estimation

After the completion of the experimental period, on the 14th day, immediately before sacrifice, the weight changes of rats were measured by subtracting the original body weight (computed on day 1) from the final body weight (measured on the last day) (Seo et al., 2018). Absolute weight change = Final Body Weight − Initial Body Weight.

### 2.5 Oral glucose tolerance test (OGTT)

The rats’ glucose tolerance was calculated using the OGTT. On day 12, rats fasted for 14 h and were then given a 40% glucose solution orally (2 g/kg body weight) (Shimoda et al., 1993; El-Sonbaty et al., 2019). The blood glucose level was assessed by collecting blood from the tail vein at 0 min (before glucose administration), 30 min, 60 min, 120 min, and 180 min. The glucose was administered after the 14-day experimental period. The blood glucose level was computed using a compact glucometer (Accu-Chek Instant, Roche Holding AG, United Kingdom).

### 2.6 Collection of biological samples

After the 14-day investigational phase, rats were fasted for 14 h and anesthetized by thiopental sodium (50 mg/kg, i.p.), and then blood and biological samples were assembled. Blood samples were collected by retro-orbital puncture, left to clot for 30 min, and then centrifuged at 3,000 × g at 4°C for another 15 min to get serum for biochemical measurements and the assay of serum lipid profile. The rats were then sacrificed, the abdomen was opened, and the liver was harvested and then cleaned with phosphate-buffered saline (PBS) and dried using tissue paper. The livers were divided into three parts: the left median lobes were homogenized in PBS using a handheld Omni-125 homogenizer (Omni International, United States), yielding 10% w/v homogenates for the detection of oxidative stress indicators and ELISA measurements. The right lobes were fixed in 10% formalin for histopathological and immunohistochemical investigations. An aortic homogenate was created by preserving the first section of the abdominal aortas in PBS at −80°C, while the second slice was preserved in neutral-buffered 10% formalin for immunohistochemical and histopathological analyses.

### 2.7 Estimation of fasting serum insulin levels

A rat insulin ELISA kit (Cat. No. RAI006R, BioVendor, Brno, Czech Republic) was used to quantify the levels of fasting insulin in sera of rats fasted for 14 h according to the manufacturer’s directions.

### 2.8 Estimation of hepatic function biomarkers in serum

Assessments of serum aspartate aminotransferase (AST), alanine aminotransferase (ALT), and lactate dehydrogenase (LDH) levels were carried out using market-presented kits (Agappe, Kerala, India, Cat. No. 11408005, 11409005, and 11407001, respectively) according to the manufacturer’s instructions.

#### 2.8.1 Estimation of serum lipid profile

Serum levels of total cholesterol (TC) and triglycerides (TG) were measured using colorimetric kits from Genesis, Obour city, Egypt, Cat. No. 1103102 and 1108102, respectively, and high-density lipoprotein cholesterol (HDL-C) was measured by kits from Biodiagnostic, Cairo, Egypt, Cat. No. HDL114100. Serum levels of low-density lipoprotein cholesterol (LDL-C) and very-low-density lipoprotein cholesterol (VLDL-C) were calculated using the Friedewald equation (Friedewald et al., 1972) LDL-C = TC − [VLDL-C + HDL-C]; VLDL-C = TG/5.

#### 2.8.2 Estimation of oxidative stress markers

Hepatic and aortic tissues were used for the measurement of malondialdehyde (MDA), superoxide dismutase (SOD), total antioxidant capacity (TAC), and glutathione (GSH) levels according to the manufacturer’s protocols (Biodiagnostic kits, Cairo, Egypt, Cat. No. MD2529, SD2521, TA2513, and GR2511, respectively).

### 2.9 ELISA assessment

NOD-like receptor family pyrin domain-containing 3 (NLRP3) was assessed using a Fine test kit (Hubei, China, Cat. No. ER 1965). The concentration of AKT in hepatic tissues was measured using an AssayGenie kit (Dublin 2, D02 VY42, Ireland, Cat. No. RTFI01064) following the constructor’s rules. NF-κB p65 (Phospho-Ser536) was measured using MyBioSource kit (Cat. No. MBS9511033, San Diego, CA, United States), and c-Jun N-terminal kinase (JNK) was measured using an AssayGenie kit (Dublin 2, D02 VY42, Ireland, Cat. No. RTFI00924). Endothelin-1 (ET-1) was measured using a Booster kit (California, United States, Cat. No. EK0952). Vascular cell adhesion molecule-1 (VCAM-1) and AKT were measured in aortic tissues using AssayGenie kits (Dublin 2, D02 VY42, Ireland, Cat. No. RTES00881 and RTFI01064, respectively). Hepatic and aortic levels of PPAR-γ and FOXO-1 were assessed using ELISA kits (AssayGenie, Dublin 2, D02 VY42, Ireland, Cat. No. RTFI01079 and RTDL00393, respectively). Hepatic and aortic levels of rat AMP-activated protein kinase alpha 1 (AMPKα1) were assessed using an ELISA kit (Cat. No. DYC3197-2, R&D Systems, Inc. Minneapolis, MN 55413, United States).

Hepatic glucose-6-phosphatase (G6Pase) was assessed using an ELISA Kit (Cat. No. ER0967, Fine test, Hubei, China). Hepatic levels of glycogen synthase kinase 3 alpha (GSK-3α) and glycogen synthase 2 (GS2) were assessed using ELISA Kits (Cat. No. NBP3-31965 and NBP3-11800, respectively, from Novus Biologicals, LLC, Centennial, CO 80112, United States.

The aortic level of PGI2 was assessed using an ELISA Kit (Cat. No. E02P0026, BlueGene Biotech, Zhoupu Town, Pudong New Area, Shanghai, China). The hepatic level of PEPCK was assessed using an ELISA kit (Cat. No. RTFI01048, AssayGenie, Dublin 2, D02 VY42, Ireland). All experimental steps were strictly performed according to the kit instructions.

### 2.10 Assessment of histopathological damage

Hepatic and aortic tissues were dissected from rats and fixed in neutral-buffered formalin for 24 h and then washed using 70% ethanol, stirred using a magnetic stirrer, and dehydrated using a serial concentration of alcohol ranging from 70% to 99%, followed by embedding in paraffin. Paraffin blocks were sliced using a rotary microtome to produce sections with 5 µm thickness. Sections were placed on glass slides, dried overnight, and stained with hematoxylin–eosin (H&E) for histopathological inspection. Histopathological details were recorded in six sections per slide, and three photos were taken per section. The pathologist was blinded to control, diseased, and treated groups, as samples were coded by groups. Relevant background material, including study design and objectives, was disclosed to the pathologist, who worked independently and utilized a semiquantitative scale to assess pathological alterations (Gibson-Corley et al., 2013). Semiquantitative counting of hepatic steatosis was assessed via histological categorizing of inflammation; steatosis was assessed by the segment of fat in liver cells: 0 = absent, 1 ≤ 25%, 2 = 25%–50%, 3 = 50%–75%, and 4 ≥ 75%, and the inflammation was rated from 0 (normal) to 3 (severe) (Abdelmageed et al., 2019). The evaluations were achieved microscopically (Leica Imaging Systems, Cambridge, United Kingdom).

Histopathological alterations in the aorta observation were assessed from 0 to 3, where 0 = nonexistence of histopathological lesions, 1 = restricted focal scattering of lesions, 2 = moderate histopathological lesions, and 3 = serious lesions on the globally observed slices (Dong et al., 1992).

### 2.11 Immunohistochemical analysis

Polyclonal antibodies were obtained from Thermo-Fisher Scientific Anatomical Pathology, California, United States, including TNF-α; Cat No. PA1-40281, IRS1; PA5-104876, eNOS; PA1-037, CD34; MA5-35202, respectively, and VEGF antibodies (Cat No. bs-0279R, Bioss, Inc., Woburn, Massachusetts, United States) for utilization in the Biotin-Avidin complex method (Guesdon et al., 1979). They were also used for immunohistochemical analysis of TNF-α and insulin receptor substrate-1 (IRS1) in both hepatic and aortic tissues and for analysis of endothelial nitric oxide synthase (eNOS), CD34, and vascular endothelial growth factor (VEGF) in aortic tissues. Slides were examined using a trinocular microscope (MBL4000-T-F-LED). The percentage of positive areas was investigated in three slices per group via ImageJ analysis software. The average percentage was computed.

### 2.12 Statistical analysis

Data are conveyed as mean ± standard error of the mean (SEM) and were examined with GraphPad Prism 8.0.2, version 8.0 software. Differences between groups means were computed by a one-way analysis of variance (ANOVA), followed by Tukey’s Kramer multiple comparisons test for parametric data. The Kruskal–Wallis test, followed by Dunn’s test, was utilized for non-parametric data, and data are expressed as median ± interquartile range (IQR). Data normality was tested by using the D’Agostino & Pearson omnibus normality test using GraphPad software Prism V 9 (GraphPad Software Inc., San Diego, CA, United States). Additionally, every possible comparison between the study groups was considered.

P values <0.05 were used to indicate statistical significance.

## 3 Results

Results revealed that the Mang CTRL group had insignificant differences from the control group regarding all measured biomarkers.

### 3.1 Impact of Mang on body weight change, OGTT_AUC_, and fasting serum insulin levels

**FIGURE 1:**
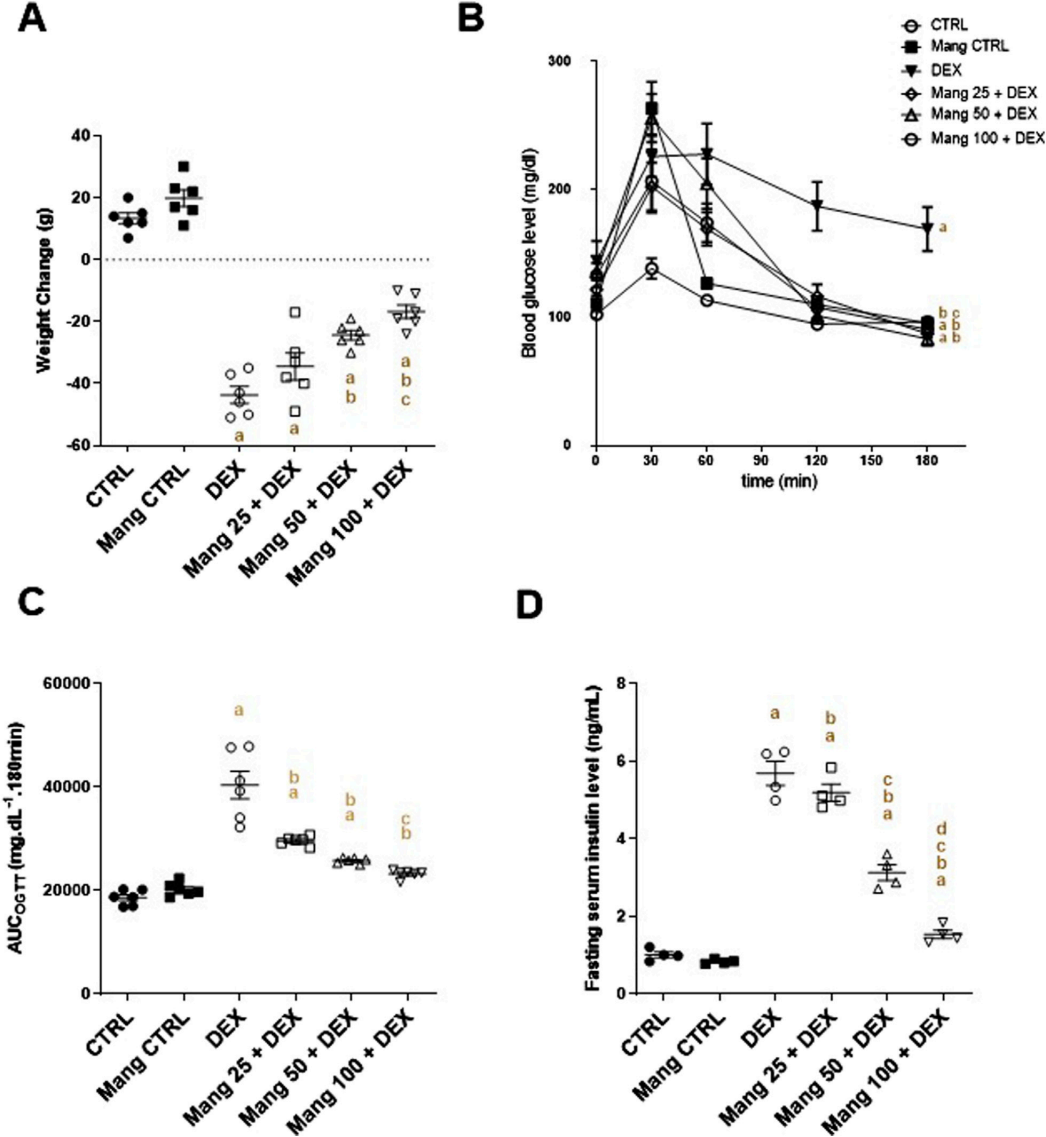
Impact of Mang on body weight change, OGTT_AUC_, and fasting serum insulin: **(A)** Body weight change. **(B)** Curve showing the changes in blood glucose. **(C)** Bar chart showing the area under the curve (AUC). **(D)** Figure showing the changes in fasting serum insulin level. AUC: Area under the curve; CTRL: Control; DEX: dexamethasone; Mang: mangiferin; OGTT: oral glucose tolerance test. Data are expressed as mean ± SEM, n = 4–6. a, b, c, d P < 0.05 compared to the CTRL, DEX, Mang 25 + DEX, Mang 50 + DEX, and Mang 100 + DEX groups, respectively, using one-way ANOVA followed by the Tukey–Kramer multiple comparisons *post hoc* test.

Animals were weighed at the start and at the end of the investigation. A significant (P < 0.05) decrease in the rats’ body weight was recorded in the DEX-injected group compared with the normal control rats. Simultaneous use of Mang (25 mg/kg, 50 mg/kg, and 100 mg/kg) mitigated DEX-induced decreases in body weight by 0.79-, 0.55-, and 0.38-fold, respectively, but it was still significantly (P < 0.05) less than the control group (Figure 1A). These results indicated a stronger effect of the higher dose of Mang (100 mg/kg) in mitigating the body weight reduction than the lower doses.

DEX-injected rats showed a glucose intolerance effect and presented a significant (P < 0.05) increase in the OGTT_AUC_ by 2.16-fold relative to the control rats. This GTT result was considerably inverted by Mang (25 mg/kg, 50 mg/kg, and 100 mg/kg), as evidenced by the respective decreases in the OGTT_AUC_ of 26.6%, 36.1%, and 42.5%, compared to the DEX group (Figures 1B,C). Fasting serum insulin was increased by 5.62-fold in the DEX group compared to the control group. Administration of Mang (25 mg/kg, 50 mg/kg, and 100 mg/kg) caused a significant (P < 0.05) decrease in fasting serum insulin of 8.78%, 44.99%, and 72.93% compared to the DEX group (Figure 1D).

These results indicated a better effect of the higher dose of Mang (100 mg/kg) in mitigating the OGTT_AUC_ and fasting serum insulin levels compared to the lower doses, but all treated groups still had a significant (P < 0.05) difference from the control group.

### 3.2 Impact of Mang on histopathological changes of hepatic tissues and serum AST, ALT, and LDH levels

**FIGURE 2:**
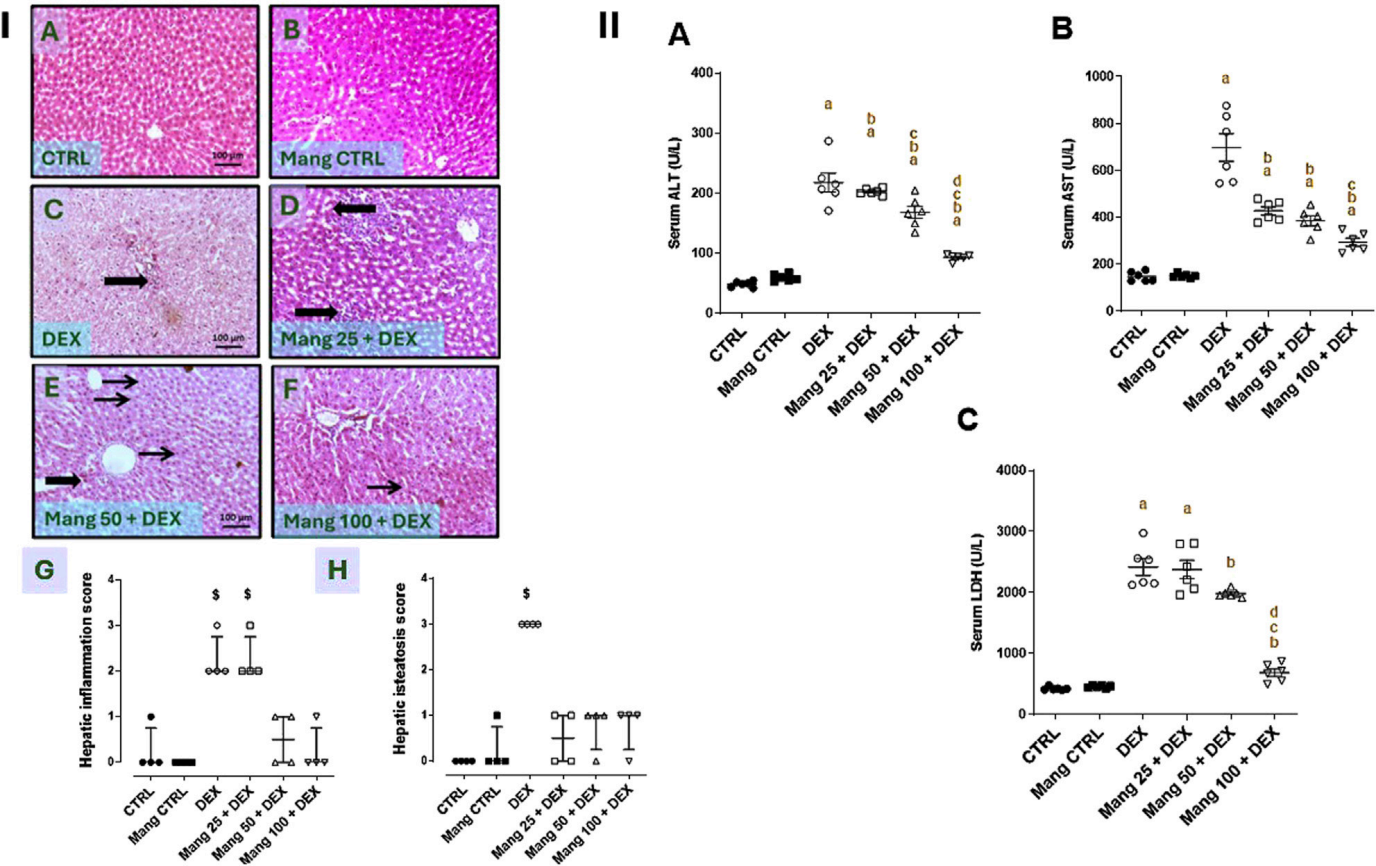
Impact of Mang on histopathological alterations of hepatic specimens and serum levels of liver function biomarkers (Panel **I**). Representative photomicrograph of livers from different treatment groups (Image magnification = ×100): **(A)** CTRL: showing normal histological appearance of hepatic parenchyma. **(B)** Mang CTRL: showing hepatic parenchyma mostly appearing normal. **(C)** DEX: showing steatohepatitis characterized by marked hepatic vacuolation (thin arrows) with mild focal periportal inflammatory aggregates beside mild perivascular fibrosis (thick arrows). **(D)** Mang 25 + DEX: showing multifocal inflammatory aggregations replacing hepatic parenchyma (thick arrows). **(E)** Mang 50 + DEX: showing occasional tiny hepatic vacuoles (thin arrow) and minimal inflammation (thick arrow). **(F)** Mang 100 + DEX: showing up to 90% of hepatic parenchyma appearing normal with few microvesicular hepatic vacuolations (thin arrow). **(G)** Hepatic inflammation score. **(H)** Hepatic steatosis score. CTRL: control; DEX: dexamethasone; Mang: mangiferin. Data are expressed as median ± interquartile range (IQR), n = 4, and were statically analyzed using the Kruskal–Wallis test followed by the Dunn multiple comparisons test (^$^ P < 0.05 vs. CTRL) (Panel **II**). Impact of Mang on serum levels of liver function biomarkers. **(A)** ALT; **(B)** AST; **(C)** LDH. CTRL: Control; DEX: dexamethasone; Mang: mangiferin; ALT: alanine aminotransferase; AST: aspartate aminotransferase; LDH: lactate dehydrogenase. Data are expressed as mean ± SEM, n = 6. a, b, c, d; P < 0.05 compared to the CTRL, DEX, Mang 25 + DEX, Mang 50 + DEX, and Mang 100 + DEX groups, respectively, using one-way ANOVA followed by the Tukey–Kramer multiple comparisons *post hoc* test.

As revealed in Figure 2I, hepatic specimens of the CTRL and Mang CTRL groups showed the normal histological appearance of the hepatic parenchyma (Figures 2IA,B). Conversely, liver specimens of the DEX group showed steatohepatitis characterized by marked hepatic vacuolation with mild focal periportal inflammatory aggregates beside mild perivascular fibrosis (Figure 2IC). The liver specimen from the Mang 25 + DEX group showed multifocal inflammatory aggregations replacing hepatic parenchyma (Figure 2ID), the liver specimen from the Mang 50 + DEX group showed occasional tiny hepatic vacuoles with minimal inflammation (Figure 2IE), and in the Mang 100 + DEX group, up to 90% of hepatic parenchyma appeared normal with few microvascular hepatic vacuolations (Figure 2IF). Figures 2IG,H show substantially greater scores of liver inflammation and steatosis in the DEX-treated rats compared to the control, but these changes are reduced in the three Mang groups, with minute differences among them.

As shown in Figure 2II, serum ALT, AST, and LDH quantities were significantly (P < 0.05) increased by 4.5-, 4.7-, and 5.71-fold, respectively, in the DEX group compared to the control group. These elevations were attenuated by Mang (25 mg/kg, 50 mg/kg, and 100 mg/kg) pretreatment, where serum ALT (Figure 2IIA) levels were decreased by 6.8%, 22.5%, and 57.2%, respectively, serum AST (Figure 2IIB) levels were decreased by 38.6%, 44.6%, and 57.8%, respectively, and LDH (Figure 2IIC) levels were decreased by 1.73%, 18.05%, and 30.3%, respectively, compared to the DEX group.

These findings revealed a better effect of the higher dose of Mang (100 mg/kg) in decreasing the AST, ALT, and LDH levels compared with the other lower doses, but all treated groups still had significant (P < 0.05) differences from the control group regarding serum ALT and AST levels.

### 3.3 Impact of Mang on serum lipid profiles

**FIGURE 3:**
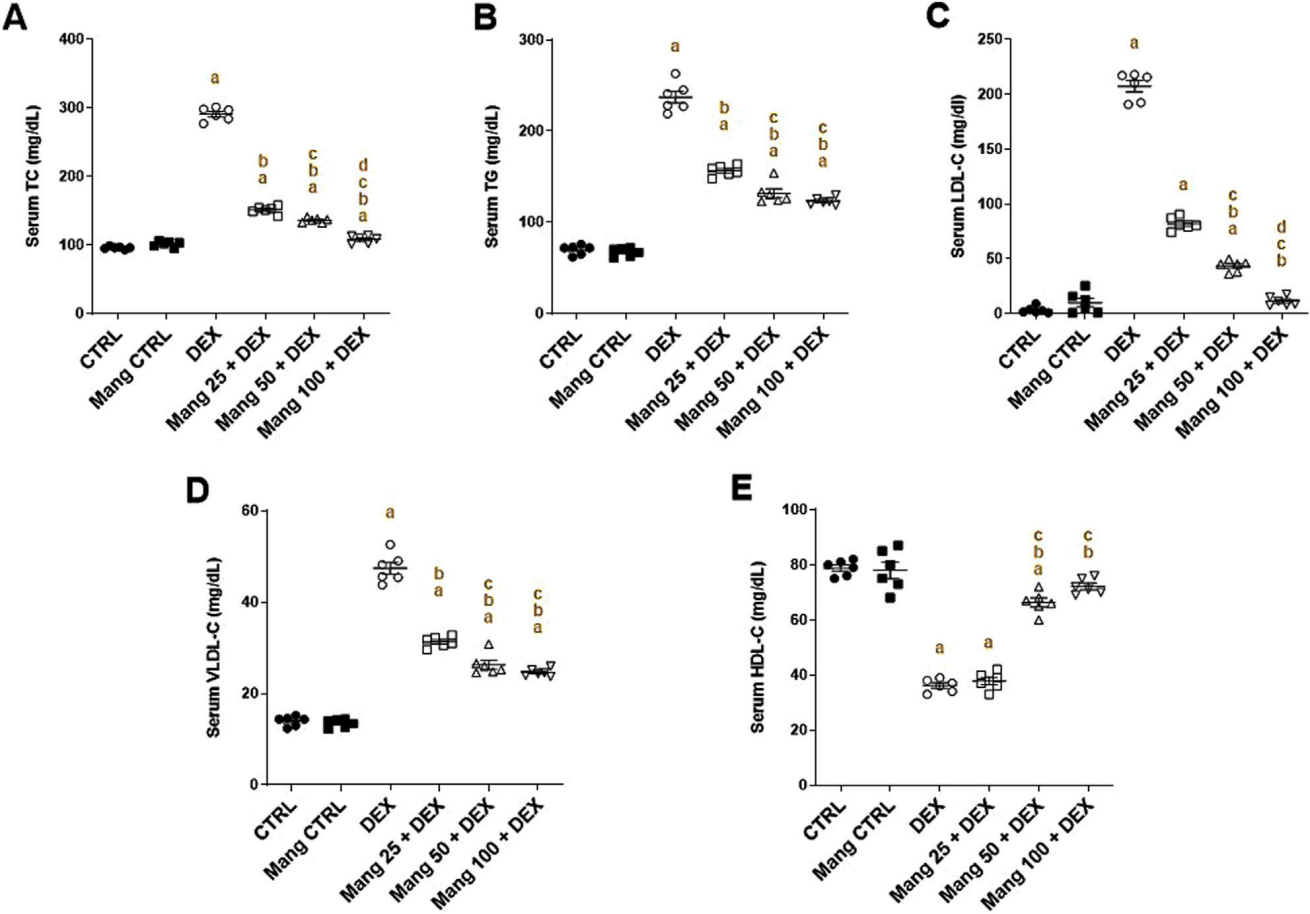
Impact of Mang on changes in the lipid profile parameters: **(A)** Serum levels of TC. **(B)** Serum levels of TG. **(C)** Serum levels of LDL-C. **(D)** Serum of levels VLDL-C. **(E)** Serum of levels HDL-C. CTRL: Control; TC: total cholesterol; TG: triglycerides; LDL-C: low-density lipoprotein; VLDL-C: very-low-density lipoprotein; HDL-C: high-density lipoprotein; DEX: dexamethasone; Mang: mangiferin. Data are expressed as mean ± SEM, n = 6. a, b, c, d; P < 0.05 compared to the CTRL, DEX, Mang 25 + DEX, Mang 50 + DEX, and Mang 100 + DEX groups, respectively, using one-way ANOVA, followed by the Tukey–Kramer multiple comparisons *post hoc* test.

As shown in Figure 3, the DEX group resulted in significant (P < 0.05) increases in TC (Figure 3A), TG (Figure 3B), LDL-C (Figure 3C), and VLDL-C (Figure 3D) by 3.03-, 3.38-, 65.4-, and 3.3-fold, respectively, compared to the control group, while serum HDL-C (Figure 3E) level decreased by 54.11%. Pretreatment with Mang (25 mg/kg, 50 mg/kg, and 100 mg/kg) resulted in an ameliorating effect versus the DEX-provoked increase in serum TC levels by 48%, 53.1%, and 62.8%, respectively, in serum TG levels by 33.9%, 44.4%, and 48%, respectively, in serum LDL-C levels by 60.46%, 79%, and 94.5%, respectively, and in serum VLDL-C levels by 33.9%, 44.4%, and 47.7%, respectively, relative to the DEX group. At the same time, serum HDL-C levels increased by 1.04-, 1.83-, and 1.99-fold compared to the DEX group.

These outcomes indicated a better effect of the larger dose of Mang (100 mg/kg) in mitigating the serum lipid profile level compared with the other lower doses, but all treated groups still had a significant (P < 0.05) difference from the control group regarding TC, TG, and VLDL-C.

### 3.4 Impact of Mang on hepatic levels of NLPR3 and TNF-α

**FIGURE 4:**
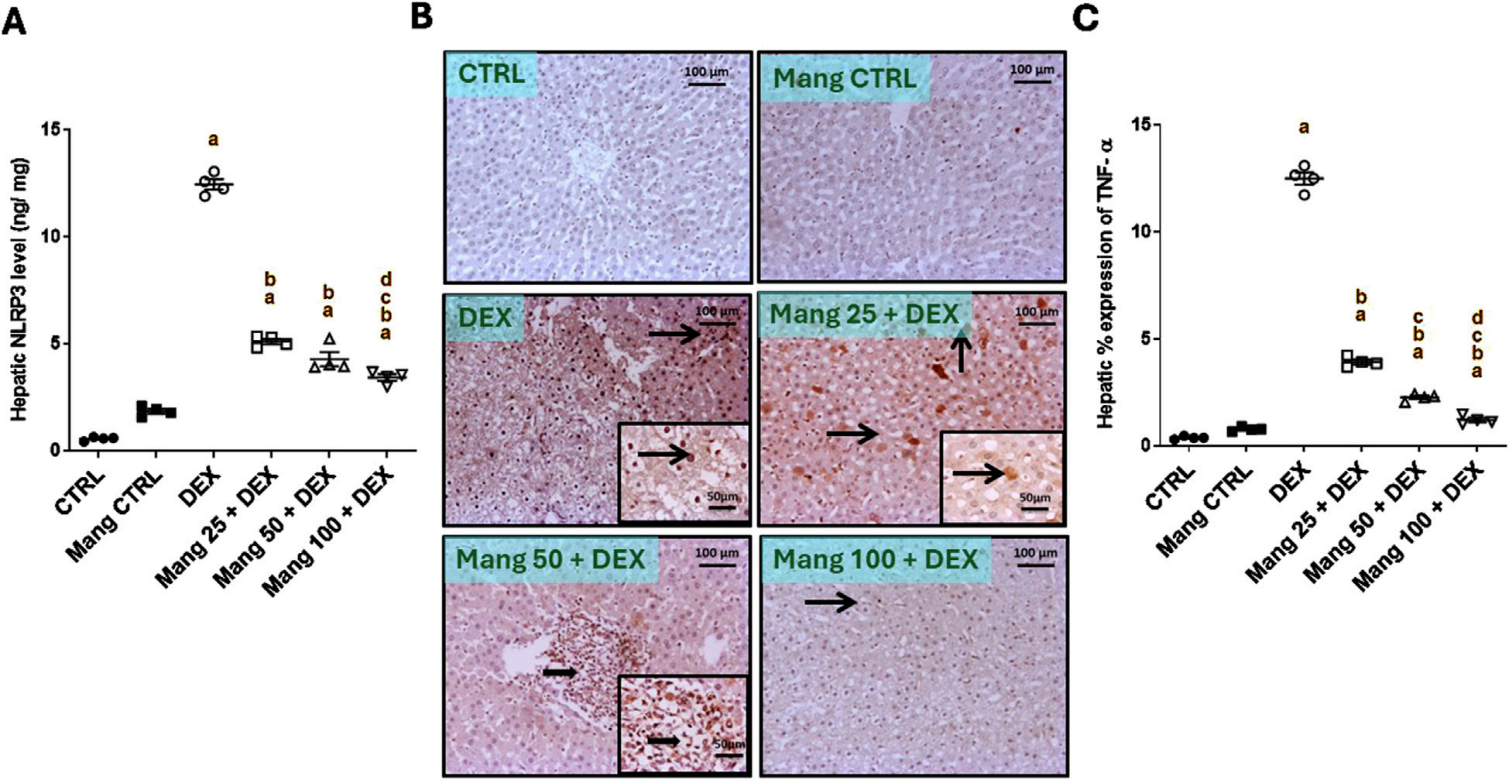
Impact of Mang on hepatic levels of NLPR3 and TNF-α: **(A)** Hepatic NLRP3 levels. **(B)** Representative IHC staining of TNF-α expression in liver sections of different treatment groups. **(C)** Hepatic % expression of TNF-α. The thin arrow indicates immunopositive stained hepatocytes, and the thick arrow = positive inflammatory cells. Image magnification = ×100, inset = ×400. CTRL: Control; NLRP3: NOD-like receptor family pyrin domain containing 3; TNF-α: tumor necrosis factor alpha; DEX: dexamethasone; Mang: mangiferin. Data are expressed as mean ± SEM, n = 4. a, b, c, d; P < 0.05 compared to the CTRL, DEX, Mang 25 + DEX, Mang 50 + DEX, and Mang 100 + DEX groups, respectively, using one-way ANOVA, followed by the Tukey–Kramer multiple comparisons *post hoc* test.

The hepatic level of NLPR3 was significantly (P < 0.05) increased in the DEX group compared to the control group by 22.56-fold, whereas Mang (25 mg/kg, 50 mg/kg, and 100 mg/kg) administration markedly lessened hepatic NLPR3 levels by 58.96%, 65.61%, and 72.56%, respectively, compared to the DEX group (Figure 4A).

The immune staining of TNF-α (Figure 4B) in hepatic specimens of the CTRL and Mang CTRL groups showed negative immunostaining in hepatic parenchyma. Meanwhile, the DEX group showed diffuse immunopositive stained hepatocytes and inflammatory cells, with nuclear and cytoplasmic expression of TNF-α in hepatocytes and cellular infiltrates. Conversely, the Mang 25 + DEX group displayed a modestly strong expression of scattered immunopositive stained hepatocytes, with intense nuclear and cytoplasmic staining of hepatocytes. A slight improvement in the Mang 50 + DEX group was shown by strong focal immunopositive staining of inflammatory aggregates. Meanwhile, the Mang 100 + DEX showed faint immunostaining hepatocytes. A statistical assessment of the immunohistochemically (IHC)-stained slide percentages of positively stained areas revealed that hepatic percent immune expression of TNF-α (Figure 4C) was significantly (P < 0.05) increased in the DEX group compared to the control group by 33.28-fold. Mang (25 mg/kg, 50 mg/kg, and 100 mg/kg) administration significantly (P < 0.05) decreased hepatic percent immune expression of TNF-α by 68.62%, 81.91%, and 90.36%, respectively, compared to the DEX group.

It was found that the ameliorative effect of Mang was dose-dependent as results indicated a better effect of the higher dose of Mang (100 mg/kg) in mitigating the NLPR3 and TNF-α levels compared with the other lower doses, but all treated groups still had a significant (P < 0.05) difference from the control group.

### 3.5 Impact of Mang on hepatic levels of IRS1 and AKT

**FIGURE 5:**
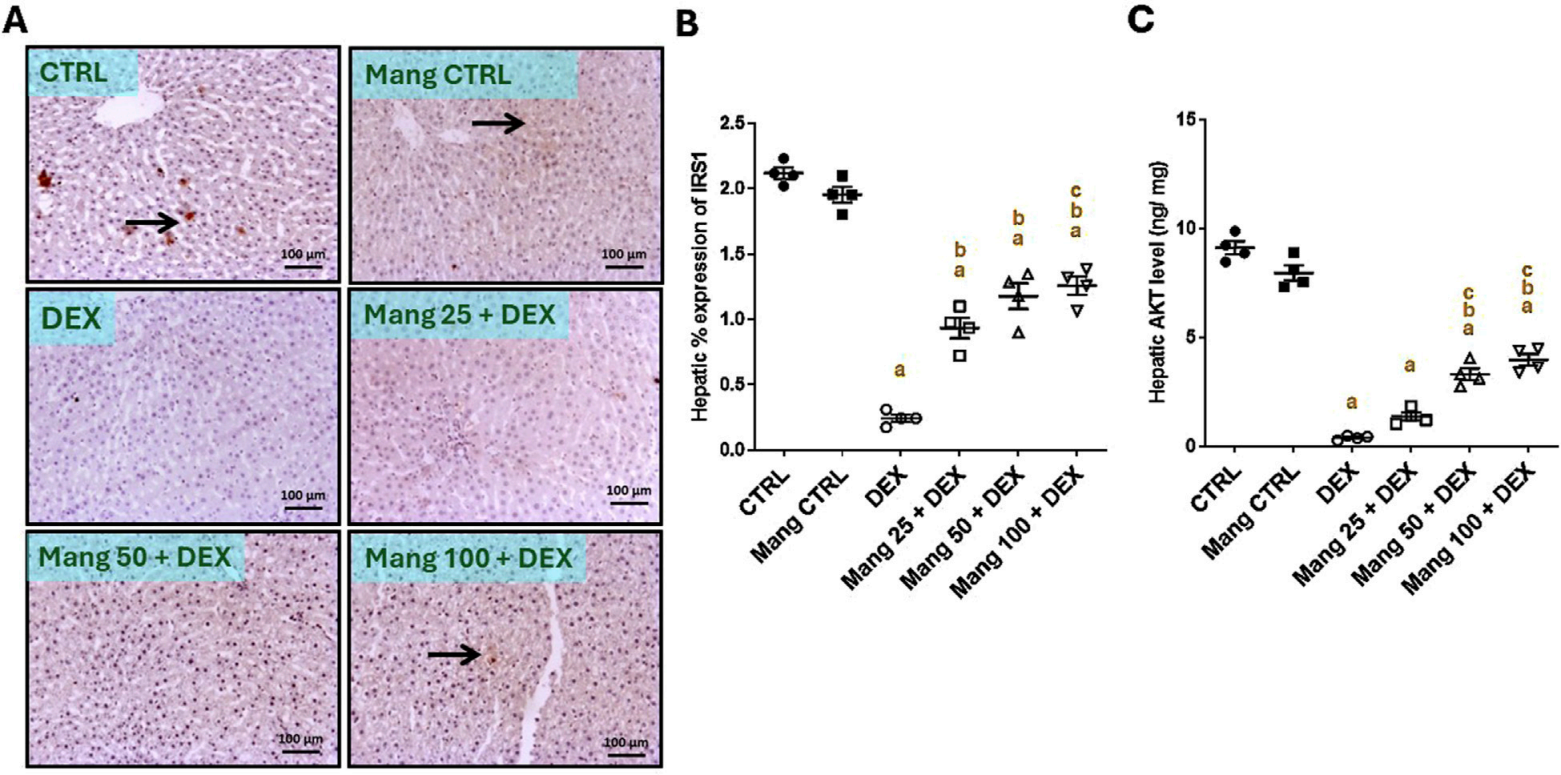
Impact of Mang on hepatic levels of IRS1 and AKT: **(A)** Representative IHC staining of IRS1 expression in liver sections of different treatment groups. **(B)** Hepatic % expression IRS-1. **(C)** Hepatic AKT level. CTRL: Control; AKT: protein kinase B; IRS1: insulin receptor substrate-1; DEX: dexamethasone; Mang: mangiferin. Data are expressed as mean ± SEM, n = 4. The thin arrow indicates immunopositive stained hepatocytes, and the thick arrow = positive inflammatory cells. Image magnification = ×100, inset = ×400. a, b, c; P < 0.05 compared to the CTRL, DEX, Mang 25 + DEX, Mang 50 + DEX, and Mang 100 + DEX groups, respectively, using one-way ANOVA followed by the Tukey–Kramer multiple comparisons *post hoc* test.

The immune staining of IRS1 (Figure 5A) in hepatic specimens of the CTRL and Mang CTRL groups showed moderate immunostaining in hepatocytes, while the DEX group showed negative to few faint immunopositive stained cells. The Mang 25 + DEX group showed faint immunostaining of hepatocytes, and the Mang 50 + DEX group showed a few immunopositive staining cells. The Mang 100 + DEX group showed mild-to-moderate expression of IRS in hepatocytes. A statistical assessment of the IHC-stained slide percentages of positively stained areas revealed that hepatic percent immune expression of IRS1 was meaningfully diminished in the DEX group compared to the control group by 88.34%. Meanwhile, Mang (25 mg/kg, 50 mg/kg, and 100 mg/kg) administration markedly improved the percentage expression of IRS1 by 2.38-, 2.62-, and 3.8-fold, compared to the DEX group (Figure 5B).

The hepatic level of AKT (Figure 5C) decreased considerably in the DEX group compared to the control group by 95.64%. Mang (25 mg/kg, 50 mg/kg, and 100 mg/kg) administration markedly increased the hepatic levels of AKT compared to the DEX group by 3.45-, 8.30-, and 9.99-fold, respectively.

These results indicated a better effect of the greater dose of Mang (100 mg/kg) in lessening the IRS1 and AKT levels compared with the other lower doses, but all treated groups still had a significant (P < 0.05) difference from the control group.

### 3.6 Impact of Mang on hepatic levels of AMPK, PPAR-γ, and FOXO-1

**FIGURE 6:**
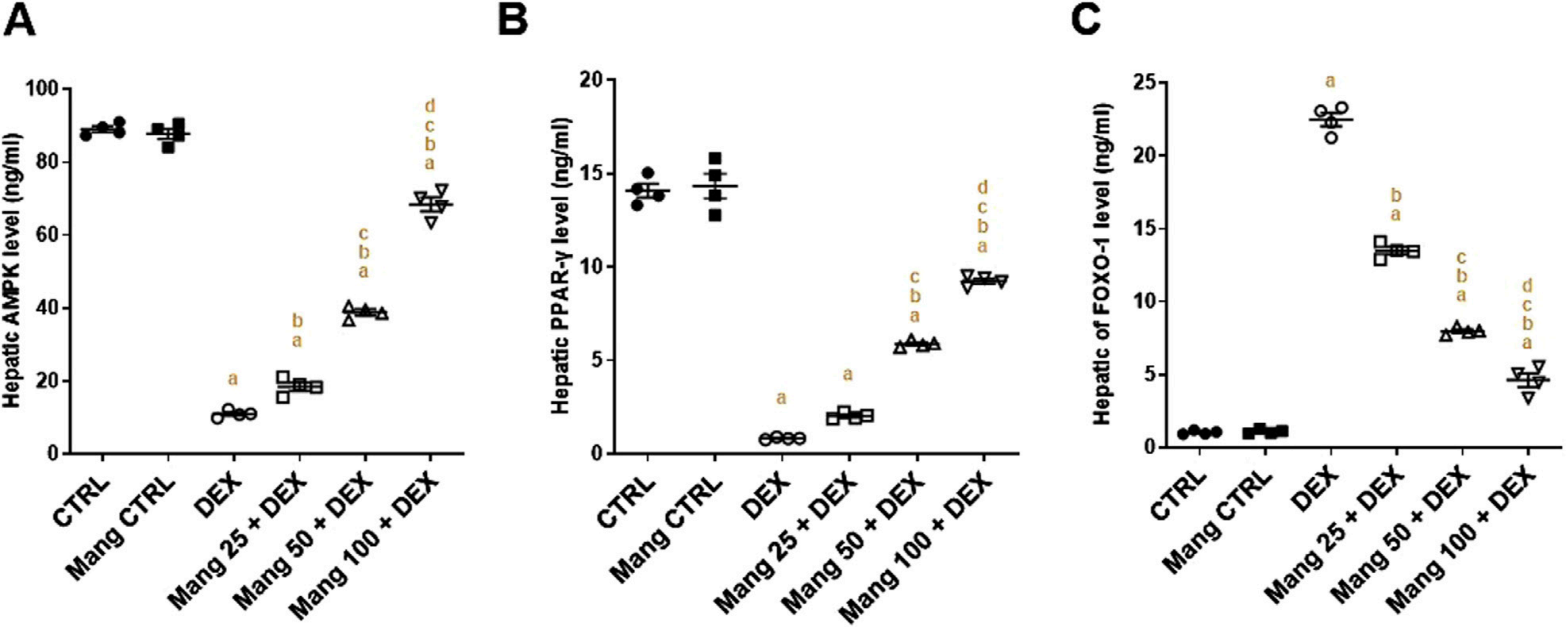
Impact of Mang on hepatic levels of AMPK, FOXO-1, and PPAR-γ: **(A)** Hepatic AMPK level. **(B)** Hepatic FOXO-1 level. **(C)** Hepatic PPAR-γ level. Data are expressed as mean ± SEM, n = 4. CTRL: control; DEX: dexamethasone; Mang: mangiferin; PPAR-γ: peroxisome proliferator-activated receptor γ; FOXO-1: forkhead box protein O1; AMPKα1: AMP-activated protein kinase alpha 1. a, b, c, d; P < 0.05 compared to the CTRL, DEX, Mang 25 + DEX, Mang 50 + DEX, and Mang 100 + DEX groups, respectively, using one-way ANOVA followed by the Tukey–Kramer multiple comparisons *post hoc* test.

The hepatic level of AMPK was significantly (P < 0.05) diminished in the DEX group compared to the control group by 87.64% (Figure 6A). Meanwhile, Mang (25 mg/kg, 50 mg/kg, and 100 mg/kg) administration markedly improved the percentage expression of AMPK by 1.68-, 3.53-, and 6.22-fold, respectively, compared to the DEX group. Additionally, the hepatic level of PPAR-γ was significantly (P < 0.05) reduced in the DEX group compared to the control group by 94.08%, whereas Mang (25 mg/kg, 50 mg/kg, and 100 mg/kg) administration markedly elevated hepatic PPAR-γ levels by 2.43-, 7.05-, and 11.08-fold, respectively, compared to the DEX group (Figure 6B).

The hepatic level of FOXO-1 was significantly (P < 0.05) increased in the DEX group compared to the control group by 21.65-fold, whereas Mang (25 mg/kg, 50 mg/kg, and 100 mg/kg) administration markedly lessened hepatic FOXO-1 levels by 39.99%, 64.50%, and 79.43%, respectively, compared to the DEX group (Figure 6C).

### 3.7 Impact of Mang on hepatic levels of GSK3α, GS2, PEPCK, and G6Pase

**FIGURE 7:**
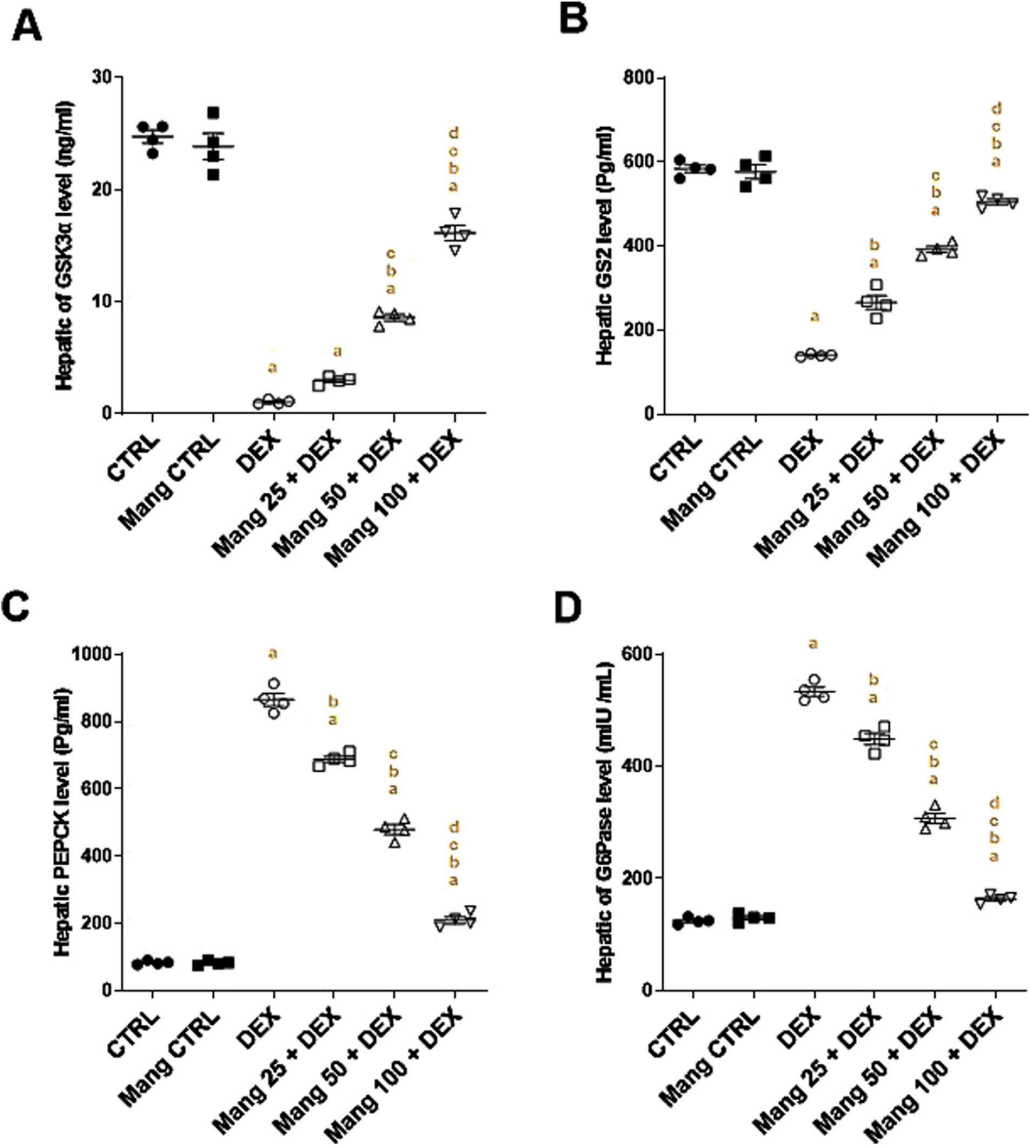
Impact of Mang on hepatic levels of GSK3α, GS2, PEPCK, and G6Pase: **(A)** Hepatic GSK3α level. **(B)** Hepatic GS2 level. **(C)** Hepatic PEPCK level. **(D)** Hepatic G6Pase level. Data are expressed as mean ± SEM, n = 4. CTRL: control; DEX: dexamethasone; Mang: mangiferin; PEPCK: phosphoenolpyruvate carboxykinase; G6Pase: glucose-6-phosphatase; GSK-3 alpha: glycogen synthase kinase 3 alpha; GS2: glycogen synthase 2. a, b, c, d; P < 0.05 compared to the CTRL, DEX, Mang 25 + DEX, Mang 50 + DEX, and Mang 100 + DEX groups, respectively, using one-way ANOVA followed by the Tukey–Kramer multiple comparisons *post hoc* test.

The hepatic level of GSK3α was significantly (P < 0.05) reduced in the DEX group compared to the control group by 95.68%, whereas Mang (25 mg/kg, 50 mg/kg, and 100 mg/kg) administration markedly elevated hepatic GSK3α levels by 2.79-, 8.05-, and 15.12-fold, respectively, compared to the DEX group (Figure 7A). Additionally, the hepatic level of GS2 was significantly (P < 0.05) reduced in the DEX group compared to the control group by 75.98%. In contrast, Mang (25 mg/kg, 50 mg/kg, and 100 mg/kg) administration markedly improved the percentage expression of GS2 by 1.89-, 2.79-, and 3.60-fold, respectively, relative to the DEX group (Figure 7B).

The hepatic level of PEPCK was significantly (P < 0.05) increased in the DEX group compared to the control group by 10.36-fold, whereas Mang (25 mg/kg, 50 mg/kg, and 100 mg/kg) administration markedly lessened hepatic PEPCK levels by 20.52%, 44.64%, and 75.71%, respectively, compared to the DEX group (Figure 7C). In addition, the hepatic level of G6Pase was significantly (P < 0.05) increased in the DEX group compared to the control group by 4.28-fold, whereas Mang (25 mg/kg, 50 mg/kg, and 100 mg/kg) administration markedly decreased hepatic G6Pase levels by 15.82%, 42.37%, and 69.32%, respectively, compared to the DEX group (Figure 9D).

### 3.8 Impact of Mang on oxidant/antioxidant parameters

**FIGURE 8:**
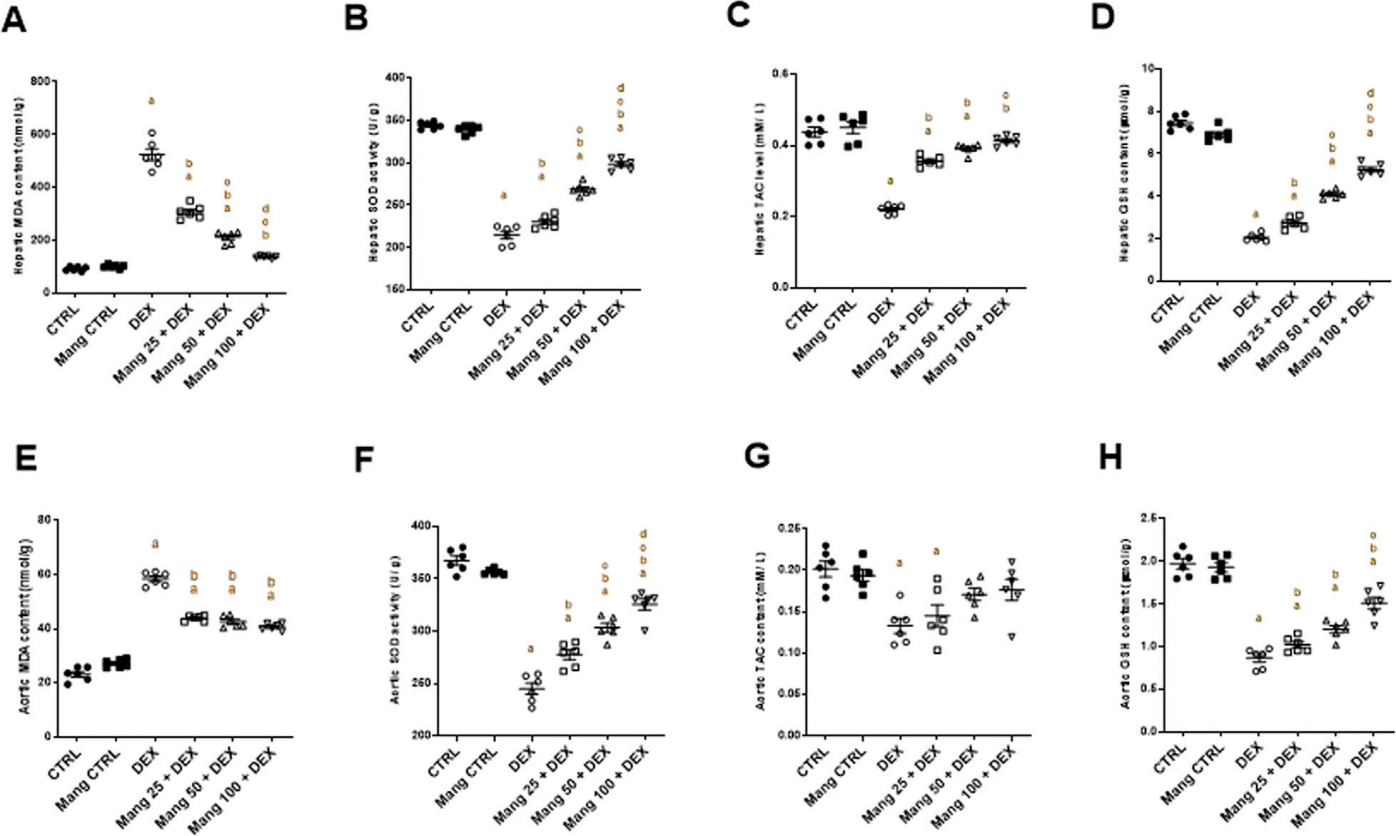
Impact of Mang on oxidative stress parameters: **(A)** Hepatic MDA content. **(B)** Hepatic SOD activity. **(C)** Hepatic TAC level. **(D)** Hepatic GSH content. **(E)** Aortic MDA content. **(F)** Aortic SOD activity. **(G)** Aortic TAC level. **(H)** Aortic GSH content. GSH: reduced glutathione; MDA: malondialdehyde; SOD: superoxide dismutase; TAC: total antioxidant capacity; CTRL: control; DEX: dexamethasone; Mang: mangiferin. Data are expressed as mean ± SEM, n = 6. a, b, c, d; P < 0.05 compared to the CTRL, DEX, Mang 25 + DEX, Mang 50 + DEX, and Mang 100 + DEX groups, respectively, using one-way ANOVA followed by the Tukey–Kramer multiple comparisons *post hoc* test.

Rats treated with DEX showed a significant (P < 0.05) increase in MDA content in both hepatic and aortic tissue by 5.63- and 2.52-fold, respectively, compared to the control group. Mang (25 mg/kg, 50 mg/kg, and 100 mg/kg) pretreatment provoked a substantial decrease in MDA levels in hepatic tissue by 41.33%, 59.67%, and 73.79%, and in aortic tissue by 25.01%, 27.20%, and 30.38%, respectively, relative to the DEX group (Figures 8A,E).

SOD activity in both hepatic and aortic tissues of the DEX group was significantly (P < 0.05) decreased by 37.63% and 33.33%, compared to the control group. Mang (25 mg/kg, 50 mg/kg, and 100 mg/kg) pretreatment prompted a significant (P < 0.05) increase in SOD levels in both hepatic tissue by 1.07-, 1.25-, and 1.38-fold and aortic tissue by 1.13-, 1.23-, and 1.32-fold, respectively, compared to the DEX group (Figures 8B,F).

Concerning TAC activity, the DEX group displayed a significant (P < 0.05) decrease in both hepatic and aortic tissue by 49.62% and 34.14%, respectively, when compared with the control group. Pre-administration with Mang (25 mg/kg, 50 mg/kg, and 100 mg/kg) resulted in considerable elevation in TAC activity in hepatic tissues by 1.61-, 1.72-, and 1.87-fold and aortic tissue homogenates by 1.09-, 1.28-, and 1.32-fold, respectively, compared to the DEX group (Figures 8C,G).

GSH levels in both hepatic and aortic tissue from the DEX group displayed a significant (P < 0.05) decrease compared to the control group by 72.45% and 56.09%; however, pretreatment with Mang (25 mg/kg, 50 mg/kg, and 100 mg/kg) resulted in an improvement in GSH activity in hepatic tissues by 1.33-, 1.99-, and 2.55-fold and aortic tissue by 1.17-, 1.38-, and 1.74-fold, respectively, compared to the DEX group (Figures 8D,H).

It was found that the ameliorative effect of Mang (100 mg/kg) mitigating the oxidative stress parameters was more significant than the other lower doses, but all treated groups still had a significant (P < 0.05) difference from the control group regarding hepatic and aortic MDA, SOD, and GSH levels.

#### 3.9 Impact of Mang on histopathological changes of aortic samplings

**FIGURE 9:**
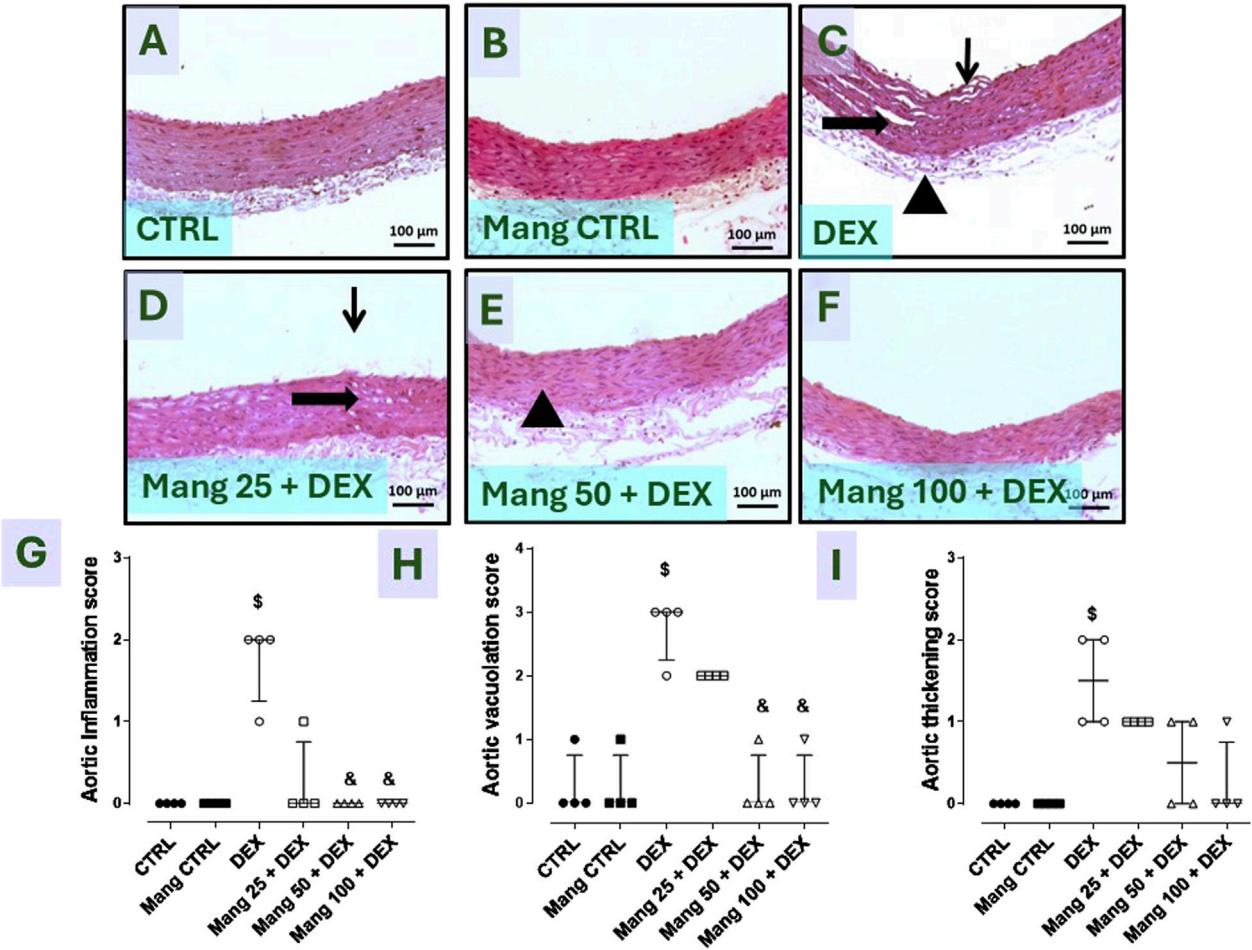
Impact of Mang on the histopathological examination of aortic specimens: Representative photomicrograph of aortic from different treatment groups: **(A)** CTRL group: showing normal layers of the aorta; **(B)** Mang CTRL group: showing normal appearance of the aortal layer; **(C)** DEX: showed intimal damage with focal luminal bulging and few invading inflammatory cells (thin arrow), vacuolation and cleft of medial layer (thick arrow), and serosal damage with minimal inflammatory invasion; **(D)** Mang 25 + DEX: showed minimal intimal bulging (thin arrow), many medial clefts and vacuolation (thick arrow); **(E)** Mang 50 + DEX: showed minimal medial invasion with inflammatory cells (arrowhead); **(F)** Mang 100 + DEX: showed up to 90% of the aortal layer appeared normal with few intimal bulging, few medial lymphocytes; **(G)** aortic inflammation score; **(H)** aortic vacuolation score; **(I)** aortic thickening score. CTRL: control; DEX: dexamethasone; Mang: mangiferin. Data are expressed as median ± IQR, n = 4, and were statically analyzed using the Kruskal–Wallis test followed by the Dunn multiple comparisons test (^$, &^ P < 0.05 vs. CTRL and DEX, respectively).

As revealed in Figure 9, aortic specimens from the CTRL and Mang CTRL groups exhibited conventional appearance without inflammation, degeneration, or necrosis (Figures 9A,B), even though the DEX group showed intimal damage with pivotal luminal bulging and a few invading inflammatory cells, vacuolation, and cleft of medial layer serosal damage with minimal inflammatory invasion (Figure 9C). In addition, the Mang 25 + DEX group showed minimal intimal bulging, many medial clefts, and vacuolation (Figure 9D), the Mang 50 + DEX group showed minimal medial invasion with inflammatory cells (Figure 9E), and the Mang 100 + DEX group showed up to 90% of the aortal layer appeared normal with little intimal bulging and few medial lymphocytes (Figure 9F).

Finally, Figures 9G–I demonstrate that the scores of aortic inflammations, vacuolation, and thickening, respectively, were meaningfully increased in the DEX group compared to the control group and decreased in the Mang (25 mg/kg, 50 mg/kg, and 100 mg/kg)-pretreated groups, with few dissimilarities amongst them.

#### 3.10 Impact of Mang on aortic levels of NF-κB and TNF-α

**FIGURE 10:**
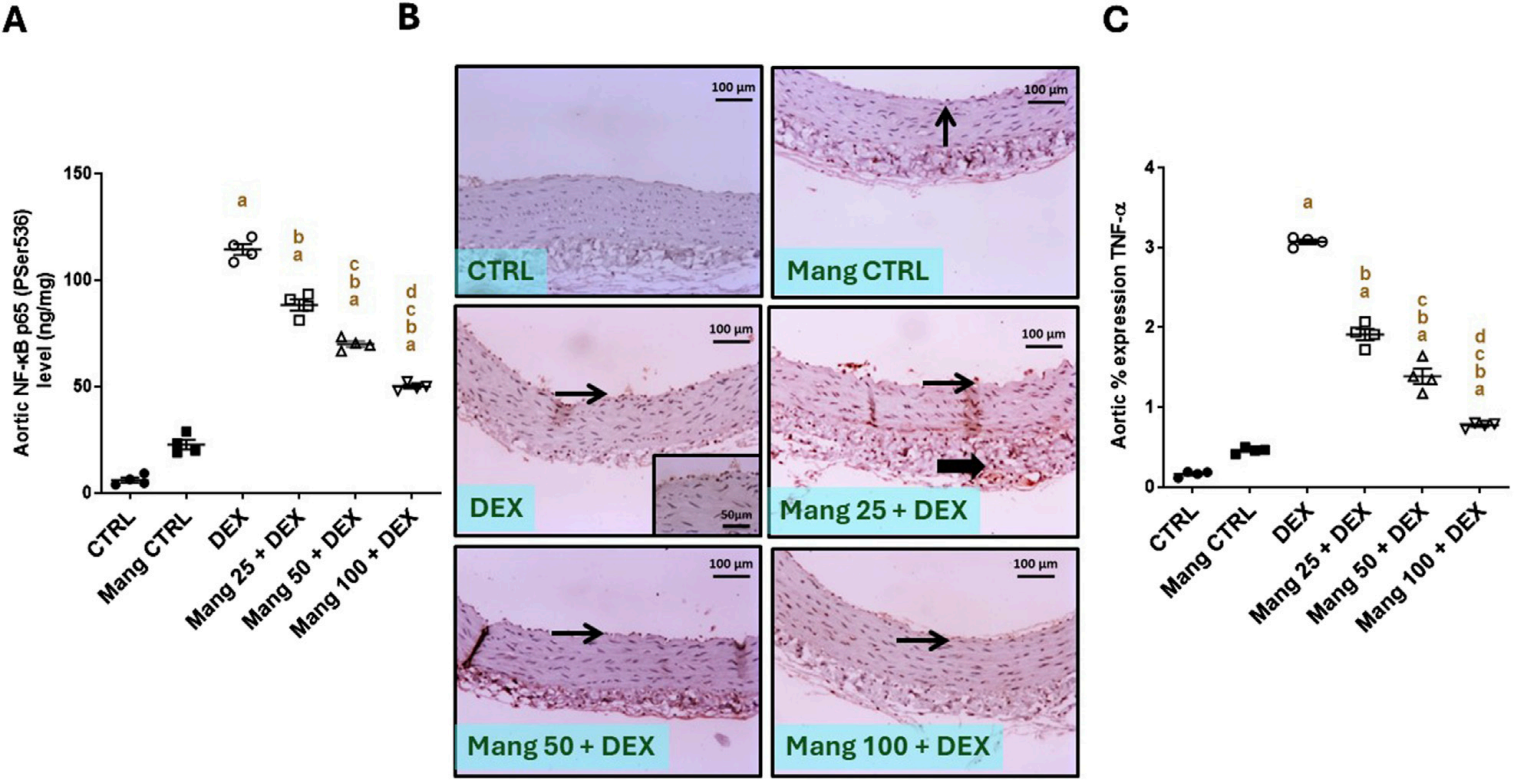
Impact of Mang on aortic levels of NF-κB and TNF-α: **(A)** aortic NF-κB P65 (P-ser536) levels. **(B)** Representative IHC staining of TNF-α expression in the aorta section of different treatment groups. **(C)** Aortic % expression TNF-α. Thin arrows indicate immunopositive-stained intimal cells; thick arrows indicate immunopositive adventitial cells. Image magnification = ×100, inset = ×400. CTRL: control; NF-κB: nuclear factor kappa B; TNF-α: tumor necrosis factor-alpha; DEX: dexamethasone; Mang: mangiferin. Data are expressed as mean ± SEM, n = 4. a, b, c, d; P < 0.05 compared to the CTRL, DEX, Mang 25 + DEX, Mang 50 + DEX, and Mang 100 + DEX groups, respectively, using one-way ANOVA, followed by the Tukey–Kramer multiple comparisons *post hoc* test.

The aortic level of NF-κB P65 (P-ser536) was significantly (P < 0.05) increased in the DEX group compared to the control group by 18.92-fold. On the other hand, Mang (25 mg/kg, 50 mg/kg, and 100 mg/kg) administration markedly decreased aortic levels of NF-κB P65 (P-ser536) compared to the DEX group by 22.73%, 38.80%, and 56.26%, respectively (Figure 10A).

The immune staining of TNF-α (Figure 10B) expression in the aortic CTRL group showed negative to few immunostainings in the intimal layer. The Mang CTRL group showed a few positively stained intimal endothelial cells. The DEX group showed moderate to high immunopositive staining in the intima and adventitia. Meanwhile, the Mang 25 + DEX group showed moderate staining in the intima and adventitia. According to our result, a slight improvement in the Mang 50 + DEX group was revealed by a mild immunopositive staining of intimal cells with a few positively stained adventitia. On the other hand, the Mang 100 + DEX group showed mild immunostaining in the intimal cells. A statistical assessment of the IHC-stained slide percentages of positively stained areas revealed that aortic percent immune expression of TNF-α (Figure 10C) was significantly (P < 0.05) increased in the DEX group compared to the control group by 18.8-fold. Mang (25 mg/kg, 50 mg/kg, and 100 mg/kg) administration markedly decreased the % TNF-α expression when compared to the DEX group by 37.83%, 74.85%, and 85.09%, respectively, but it still did not reach normal levels.

It was found that the ameliorative effect of Mang was dose-dependent as results indicated a better effect of the largest dose of Mang (100 mg/kg) in reducing the aortic NF-κB and TNF-α levels compared with the other lower doses, but all treated groups still had a significant (P < 0.05) difference from the control group. The Mang CTRL group had insignificant differences from the control group regarding serum levels of NF-κB and TNF-α.

#### 3.11 Impact of Mang on aortic levels of IRS1 and AKT

**FIGURE 11:**
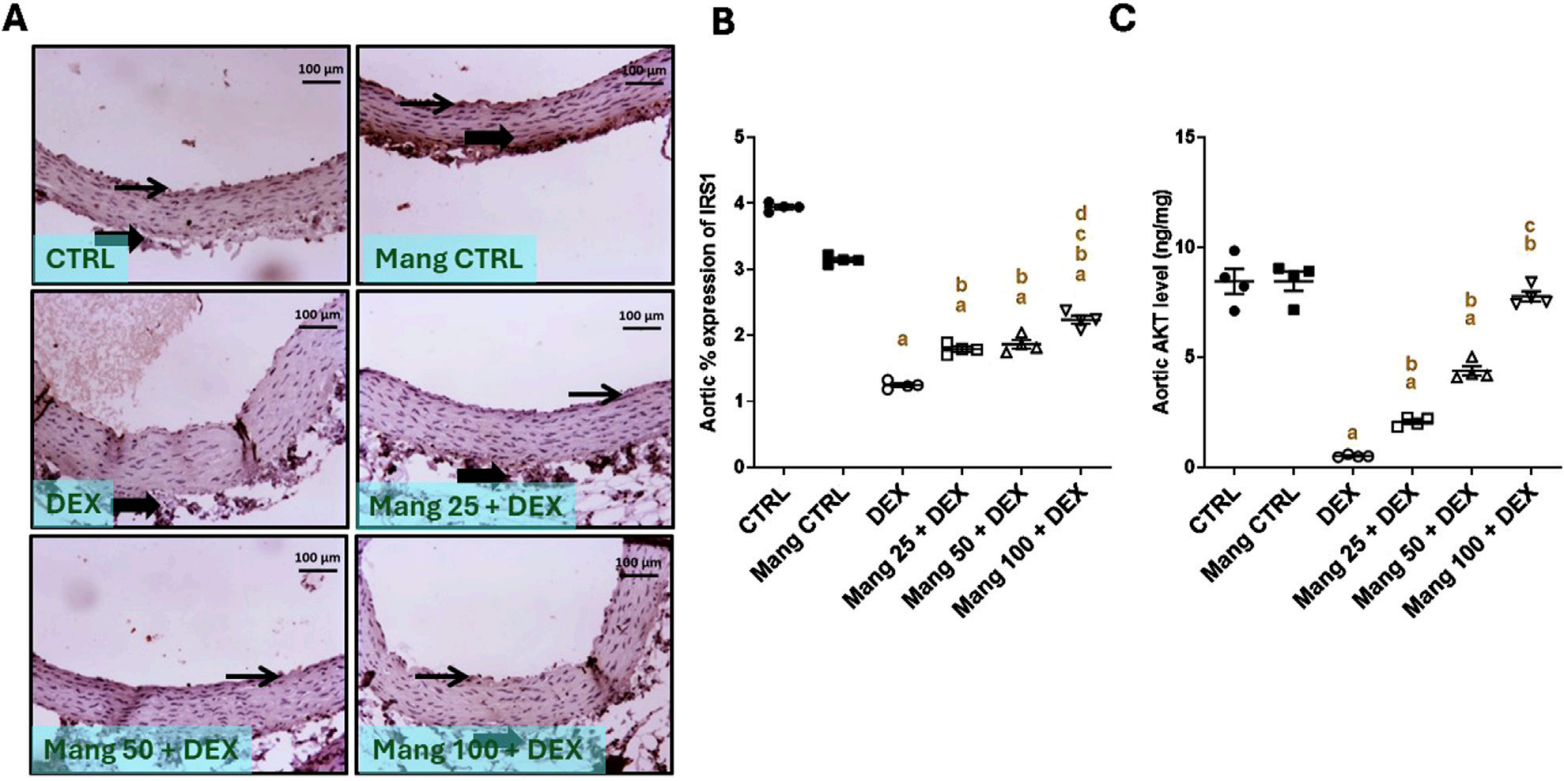
Impact of Mang on aortic levels of levels of IRS1 and AKT: **(A)** Representative IHC staining of IRS1 expression in aorta section of different treatment groups. **(B)** Aortic % expression IRS1. **(C)** Aortic AKT levels. CTRL: control; IRS1: insulin receptor substrate 1; AKT: protein kinase B; DEX: dexamethasone; Mang: mangiferin. Data are expressed as mean ± SEM, n = 4. Thin arrows indicate immunopositive-stained intimal cells. Image magnification = ×100, inset = ×400. a, b, c, d; P < 0.05 compared to the CTRL, DEX, Mang 25 + DEX, Mang 50 + DEX, and Mang 100 + DEX groups, respectively, using one-way ANOVA followed by the Tukey–Kramer multiple comparisons *post hoc* test.

The immune staining of IRS1 (Figure 11A) expression in the aortic CTRL group showed moderate intense immunostaining of intimal and adventitial layers, and the Mang CTRL group showed moderate to high immunostaining of intimal endothelial cells and adventitial cells. The DEX group showed a few immunopositively stained adventitial invading cells. The Mang 25 + DEX group showed mild expression of intimal endothelial cells with little positivity in adventitial cells. The Mang 50 + DEX group showed modest immunopositive staining of intimal endothelial cells. On the other hand, the Mang 100 + DEX group showed moderate immunostaining of intimal cells.

A statistical assessment of the IHC-stained slide percentages of positively stained areas (Figure 11B) revealed a significant (P < 0.05) reduction in IRS1 expression in the DEX group by 71.88% compared to the control group. Whereas Mang (25 mg/kg, 50 mg/kg, and 100 mg/kg) administration markedly increased the percentage expression of IRS1 by 1.04-, 1.24-, and 1.75-fold, respectively, compared to the DEX group.

Aortic AKT level was significantly (P < 0.05) decreased in the DEX group compared to the control group by 93.87%. Mang (25 mg/kg, 50 mg/kg, and 100 mg/kg) administration markedly increased the aortic level of AKT compared to the DEX group by 4.01-, 8.46-, and 15-fold, respectively (Figure 11C). It was found that the ameliorative effect of Mang was dose-dependent as results indicated a better effect of the largest dose of Mang in mitigating the IRS1 and AKT levels compared with the other lower doses, but all treated groups still had a significant (P < 0.05) difference from the control group regarding IRS1 and even low doses of Mang (25 mg/kg and 50 mg/kg) had a significant (P < 0.05) difference from control group regarding AKT.

#### 3.12 Impact of Mang on aortic levels of JNK, ET-1, VCAM, AMPK, PPAR-γ and FOXO-1

**FIGURE 12:**
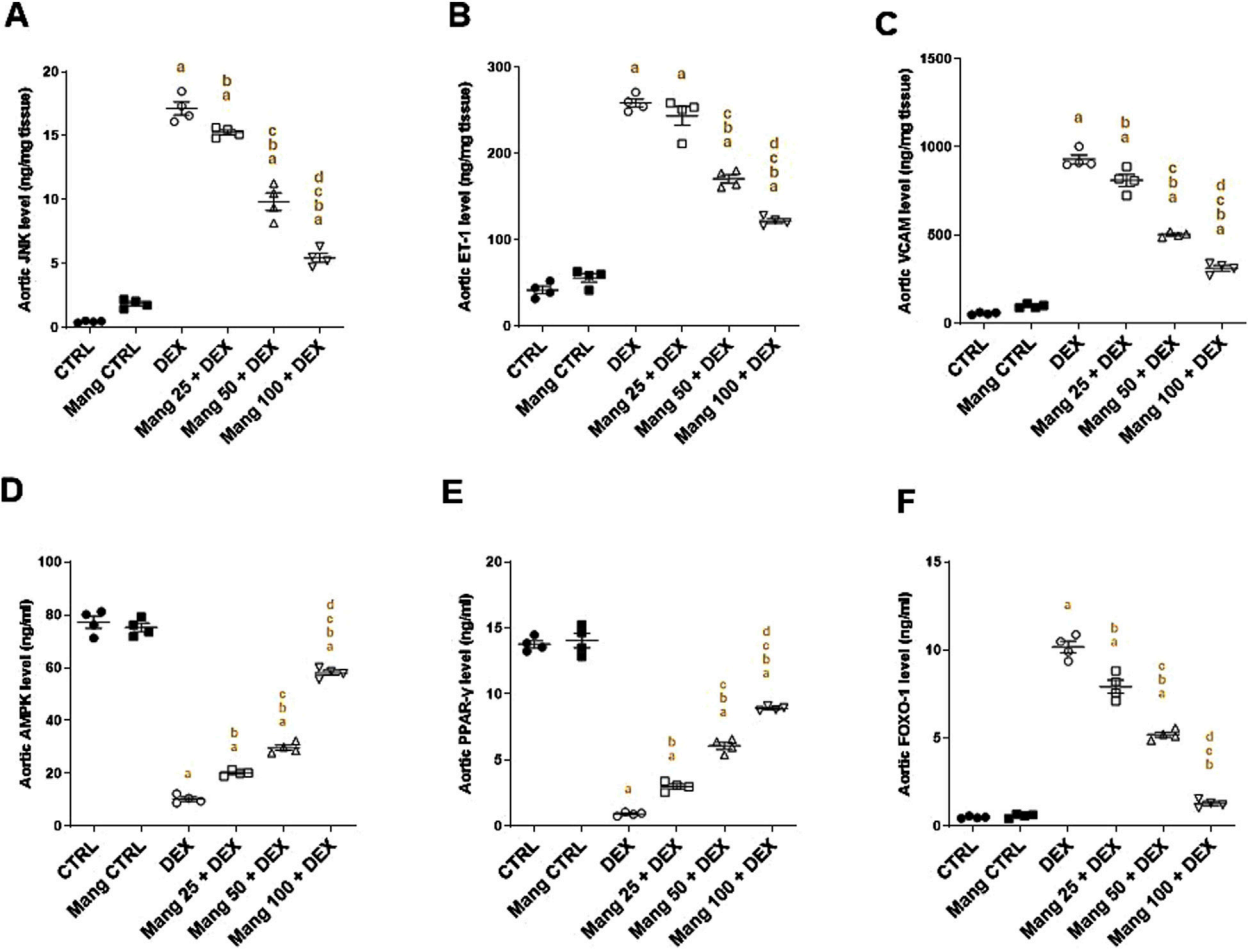
Impact of Mang on aortic levels of JNK, ET-1, VCAM, AMPK, PPAR-γ, and FOXO-1: **(A)** aortic JNK level; **(B)** aortic ET-1 level; **(C)** aortic VCAM level; **(D)** aortic AMPKα1 level; **(E)** aortic PPAR-γ level; **(F)** aortic FOXO-1 level. CTRL: Control; JNK: C-Jun N-terminal kinase; ET-1: endothelin-1; VCAM: vascular cell adhesion molecule; DEX: dexamethasone; Mang: mangiferin; PPAR-γ: peroxisome proliferator-activated receptor γ; FOXO-1: forkhead box protein O1; AMPKα1: AMP-activated protein kinase alpha 1. Data are expressed as mean ± SEM, n = 4. a, b, c, d; P < 0.05 compared to the CTRL, DEX, Mang 25 + DEX, Mang 50 + DEX, and Mang 100 + DEX groups, respectively, using one-way ANOVA followed by the Tukey–Kramer multiple comparisons *post hoc* test.

The aortic level of JNK (Figure 12A) was significantly (P < 0.05) increased in the DEX group compared to the control group by 36.29-fold. Mang (25 mg/kg, 50 mg/kg, and 100 mg/kg) administration markedly decreased aortic JNK levels by 10.85%, 42.53%, and 68.05%, respectively, compared to the DEX group. Additionally, the aortic levels of ET-1 (Figure 12B) and VCAM (Figure 12C) were significantly (P < 0.05) increased in the DEX group by 15.22- and 16.52-fold compared to the control group. Conversely, Mang (25 mg/kg, 50 mg/kg, and 100 mg/kg) administration significantly (P < 0.05) decreased aortic levels of ET-1 by 5.79%, 33.97%, and 52.80% and VCAM by 12.79%, 45.90%, and 66.38%, respectively, compared to the DEX group. The ameliorative effect of Mang was found to be dose dependent as results indicated a better effect of the largest dose of Mang (100 mg/kg) in mitigating the JNK, ET-1, and VCAM levels compared with the other lower doses, but all treated groups still had a significant (P < 0.05) difference from the control group.

The aortic levels of AMPK (Figure 12D) and PPAR-γ (Figure 12E) were significantly (P < 0.05) decreased in the DEX group compared to the control by 86.71% and 93.31%, respectively. Mang (25 mg/kg, 50 mg/kg, and 100 mg/kg) administration markedly increased aortic levels of AMPK and PPAR-γ by 1.95-, 2.89-, and 5.66-fold and 3.25-, 6.59-, and 9.66-fold, respectively, compared to the DEX group. Additionally, the aortic level of FOXO-1 was significantly (P < 0.05) increased in the DEX group compared to the control group by 20.26-fold (Figure 12F). Mang (25 mg/kg, 50 mg/kg, and 100 mg/kg) administration markedly decreased aortic FOXO-1 levels by 22%, 48.88%, and 87.42%, respectively, compared to the DEX group.

#### 3.13 Effect of Mang on aortic levels of eNOS and PGI2

**FIGURE 13:**
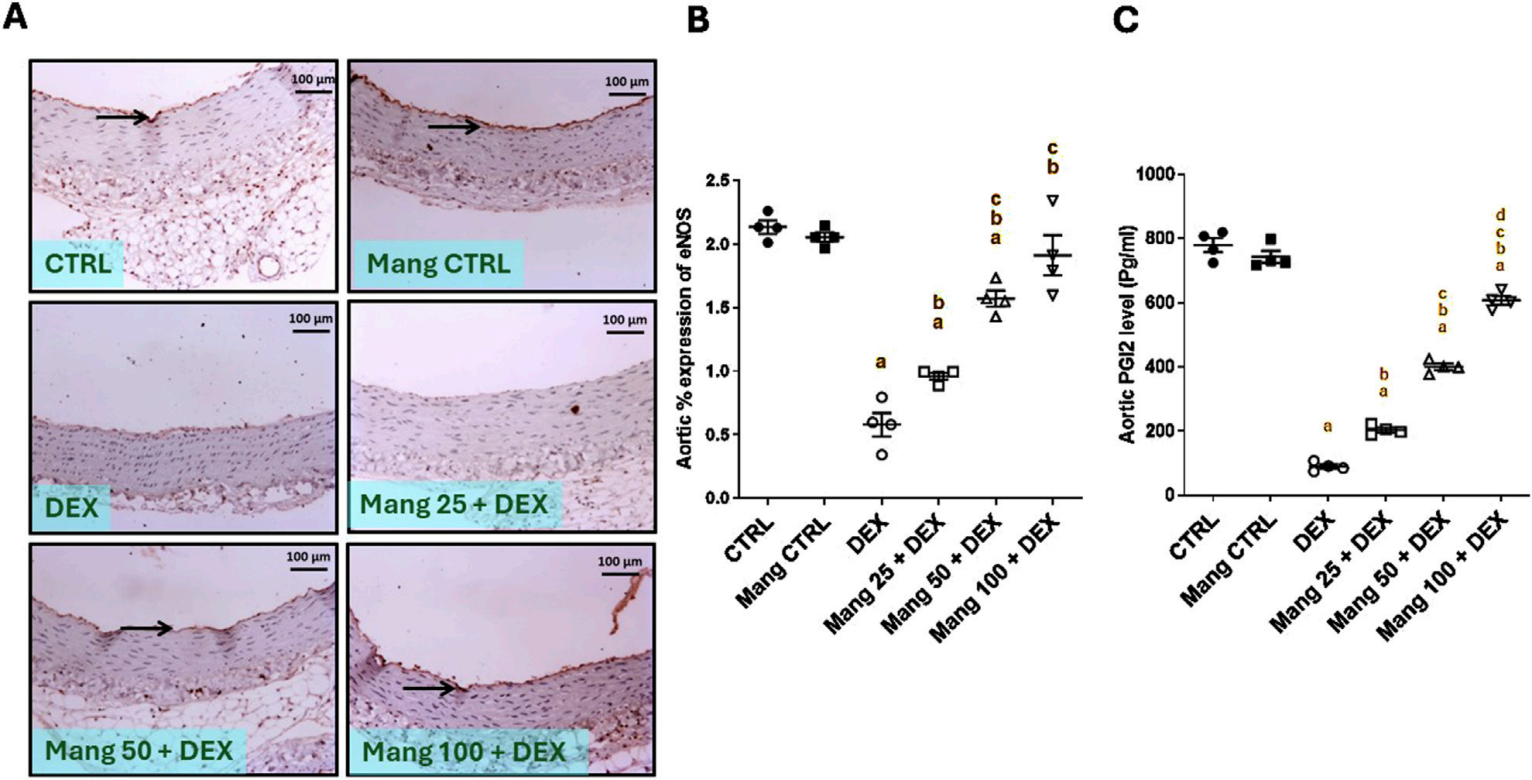
Effect of Mang on aortic levels of eNOS and PGI2: **(A)** Representative IHC staining of eNOS expression in the aorta section of different treatment groups. **(B)** Hepatic % expression eNOS. **(C)** Aortic PGI2 levels. Thin arrows indicate immunopositive stained intimal cells. Image magnification = ×100, inset = ×400. CTRL: Control; eNOS: endothelial nitric oxide synthase; DEX: dexamethasone; Mang: mangiferin; PGI2: prostacyclin. Data are expressed as mean ± SEM, n = 4. a, b, c P < 0.05 compared to the CTRL, DEX, Mang 25 + DEX, Mang 50 + DEX, and Mang 100 + DEX groups, respectively, using one-way ANOVA followed by the Tukey–Kramer multiple comparisons *post hoc* test.

According to the results, representative IHC staining of eNOS (Figure 13A) expression in the aortic CTRL group showed high immunostaining of intimal endothelial cells, and the Mang CTRL group showed high intense immunostaining of the intimal layer. The DEX group showed mild immunopositive stained endothelial cells. The Mang 25 + DEX group showed no to minimal immunopositive staining of intimal endothelial cells. The Mang 50 + DEX group showed moderate expression of intimal endothelial cells. The Mang 100 + DEX group showed moderate-to-high immunostaining intimal cells.

A statistical assessment of the IHC-stained slide percentages of positively stained areas (Figure 13B) revealed that eNOS was significantly (P < 0.05) decreased in the DEX group compared to the control group by 72.88%. Mang (25 mg/kg, 50 mg/kg, and 100 mg/kg) administration markedly increased the aortic percent expression of eNOS compared to the DEX group by 1.65-, 2.71-, and 3.30-fold, respectively. These results indicated a better effect of the largest dose of Mang (100 mg/kg) in mitigating the eNOS % expression compared with the lower doses, and even low doses of Mang (25 and 50 mg/kg) still had a significant (P < 0.05) difference from the control group.

The aortic level of PGl2 was significantly (P < 0.05) decreased in the DEX group compared to the control by 88.50% (Figure 13C). Mang (25 mg/kg, 50 mg/kg, and 100 mg/kg) administration markedly increased aortic levels of PGl2 compared to the DEX group by 2.27-, 4.46-, and 6.78-fold, respectively.

#### 3.14 Impact of Mang on the aortic expression of CD34 and VEGF

**FIGURE 14:**
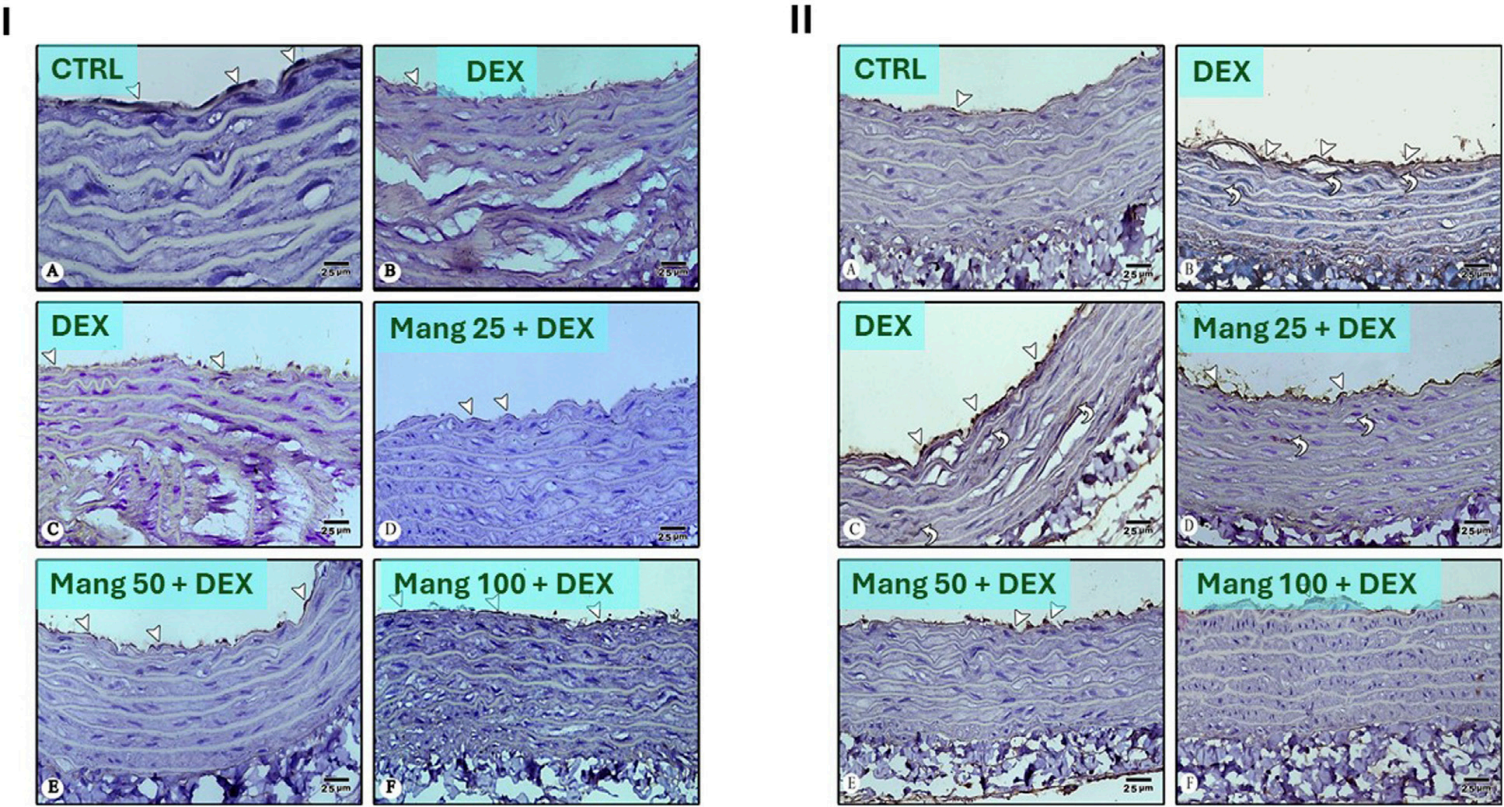
Impact of Mang on the aortic expression of CD34 and VEGF: Panel **(I)**: Photomicrographs of CD34-immune stained sections in the aorta: **(A)** CTRL group: showing strong positive CD34 immunostaining confined to the cytoplasm of endothelial cells (arrowheads). **(B,C)** DEX-treated group: showing weak positive CD34 immunostaining confined to the cytoplasm of endothelial cells (arrowheads) compared to the CTRL group. **(D)** Mang (25 mg/kg)-pretreated group: showing mild positive CD34 immunostaining confined to the cytoplasm of endothelial cells (arrowheads) but more than the DEX-treated group. **(E)** Mang (50 mg/kg)-pretreated group: showing moderate positive CD34 immunostaining confined to the cytoplasm of endothelial cells (arrowheads). **(F)** Mang (100 mg/kg)-pretreated group: showing strong positive CD34 immunostaining confined to the cytoplasm of endothelial cells (arrowheads) more or less similar to the CTRL group. Panel **(II)**: Photomicrographs of VEGF-stained sections in the aorta: **(A)** CTRL group: showing minimal VEGF immunostaining in the cytoplasm of endothelial cells (arrowhead). **(B,C)** DEX-treated group: showing more abundant positive VEGF immunostaining in the cytoplasm of endothelial cells (arrowheads) and in vascular smooth muscles (curved arrows) more than CTRL group. **(D)** Mang (25 mg/kg)-pretreated group: showing strong positive VEGF immunostaining in the cytoplasm of endothelial cells (arrowheads) and in vascular smooth muscles (curved arrows) but less than the DEX-treated group. **(E)** Mang (50 mg/kg)-pretreated group: showing moderate positive VEGF immunostaining confined to the cytoplasm of endothelial cells (arrowheads). **(F)** Mang (100 mg/kg)-pretreated group: showing scarce positive VEGF immunostaining confined to the cytoplasm of endothelial cells (arrowheads) more or less similar to the CTRL group. Image magnification = ×400. CTRL: control; DEX: dexamethasone; Mang: mangiferin; VEGF: vascular endothelial growth factor.

As shown in Figure 14I, photomicrographs of CD34-immune stained sections in the aorta revealed that the CTRL group showed strong positive CD34 immunostaining confined to the cytoplasm of endothelial cells (Figure 14IA). The DEX group showed weakly positive CD34 immunostaining confined to the cytoplasm of endothelial cells as compared to the CTRL group (Figures 14IB,C). Meanwhile, the Mang (25 mg/kg)-pretreated group showed mildly positive CD34 immunostaining confined to the cytoplasm of endothelial cells but more than the DEX-treated group (Figure 14ID). The Mang (50 mg/kg)-pretreated group showed moderately positive CD34 immunostaining confined to the cytoplasm of endothelial cells (Figure 14IE), and the Mang (100 mg/kg)-pretreated group showed strongly positive CD34 immunostaining confined to the cytoplasm of endothelial cells, more or less similar to the CTRL group (Figure 14IF).

As shown in Figure 14II, photomicrographs of VEGF-stained sections in the aorta revealed that the CTRL group showed minimal VEGF immunostaining in the cytoplasm of endothelial cells (Figure 14IIA). The DEX-treated group showed more abundantly positive VEGF immunostaining in the cytoplasm of endothelial cells and in vascular smooth muscles more than the CTRL group (Figures 14IIB,C); on the other hand, the Mang (25 mg/kg) pretreated group showed strongly positive VEGF immunostaining in the cytoplasm of endothelial cells and in vascular smooth muscles but less than the DEX-treated group (Figure 14IID), and the Mang (50 mg/kg) pretreated group showed moderately positive VEGF immunostaining confined to the cytoplasm of endothelial cells (Figure 14IIE). The Mang (100 mg/kg) pretreated group showed scarcely positive VEGF immunostaining confined to the cytoplasm of endothelial cells, which is more or less similar to the CTRL group (Figure 14IIF).

### 4 Discussion

IR is a potential hazard to human health (Liu et al., 2020). Endothelial dysfunction and IR, including IR-related morbidities such as metabolic syndrome, T2D, and obesity, are linked in a complex and reciprocal manner. Therefore, IR is associated with increased susceptibility to atherosclerotic-related cardiovascular and cerebrovascular complications, such as cerebral stroke and coronary artery disease (Gómez-Hernández et al., 2021; Sánchez et al., 2022). Our study supported the fact that i.p. administration of DEX induced IR, as evidenced by significant hyperinsulinemia, hyperglycemia, and hyperlipidemia in rats with marked lipid deposition in the aorta and liver. Mang administration was able to prevent endothelial dysfunction and hepatic steatosis using three different dose levels (25 mg/kg, 50 mg/kg, and 100 mg/kg).

As previously observed by Rafacho et al. (2010), our DEX treatment can significantly reduce body weight, an effect that was reversed by Mang administration, which showed improvement and stability in body weight. In addition, our results proved that DEX-induced central IR results in high levels of glucose and fasting serum insulin and impaired OGTT, and Mang administration suppressed DEX-induced increases in OGTT and fasting serum insulin levels after oral glucose administration. This is consistent with previous studies in which Mang has improved dyslipidemia, insulin sensitivity, and reverted adipokine levels in a rat model of T2D induced by streptozotocin (STZ) (Saleh et al., 2014). Additionally, Mang lowered blood glucose levels in KK-y mice (Miura et al., 2001a) and reversed the elevated plasma insulin and fasting plasma non-esterified fatty acid concentrations during an OGTT test in a rat model of fructose-induced metabolic syndrome (Zhou et al., 2016).

The results of this study showed that DEX administration caused acute hepatitis because of hepatic IR induction, as evidenced by increased levels of ALT, AST, and LDH, which was further confirmed by the abnormal hepatic architecture compared to normal rats. Excess glucocorticoids have been demonstrated to aid in the formation of fatty liver, and the underlying mechanisms may be linked to compensatory hyperinsulinemia and IR (Diamant and Shafrir, 1975; D'Souza A et al., 2012). It has been demonstrated that DEX stimulates insulin-induced lipogenesis in rat hepatocytes (Amatruda et al., 1983). The results of the present study showed DEX injection resulted in marked elevations in the serum levels of TC, TG, and VLDL-C and decreased HDL-C levels compared to the CTRL group. On the other hand, treatment with Mang demonstrated a dose-dependent protective effect on liver function by reducing serum ALT, AST, LDH, TC, TG, and VLDL-C levels and was able to restore the normal liver architecture and elevated HDL-C levels compared to the DEX group. This aligns with a previous study, which revealed that Mang reduced ALT and AST levels in mice and improved the liver injury induced by carbon tetrachloride (Zhang et al., 2023). In addition, Mang in STZ diabetic rats can exert potent antihyperlipidemic effects, as it maintains the homeostasis of glucolipid metabolism (Muruganandan et al., 2005).

IRS is an important molecule in the PI3K/AKT signaling pathway. DEX induces IR through numerous mechanisms, including the downregulation of IRS1 expression and function, which can negatively affect hepatic insulin signaling and exacerbate metabolic dysregulation (Geer et al., 2014). Mang-treated groups showed moderate expression of IRS1 in hepatocytes compared to the DEX group. Mang improves glucose metabolism and restores IRS1/AKT function, which in turn improves insulin sensitivity by enhancing glucose tolerance in diabetic rats, which was linked to lower inflammation and higher IRS1 expression (Yang et al., 2017). Mang successfully stopped the downregulation of the AKT signaling pathway in the kidneys of rats with STZ-induced diabetic nephropathy (Zhao et al., 2023).

One mechanism by which AKT might contribute to the insulin-mediated suppression of glycogenolysis is by driving glycogen synthesis through the activation of GS. It has been demonstrated extensively that an AKT-mediated inactivation of GSK3 contributes to a reduction in the net phosphorylation and, subsequently, activation of GS (Kvandová et al., 2016). Such an effect was proved by our data as DEX-injected rats showed marked activation of GS and GSK-3 hepatic content, indicating increased glucose production and decreased uptake as a result of IR. Such an effect was reversed by Mang administration. Furthermore, it is known that the first site of regulation of gluconeogenesis is the conversion of oxaloacetate to phosphoenolpyruvate, which is catalyzed by the enzyme PEPCK, a key rate-controlling enzyme in the pathway of gluconeogenesis (Sun et al., 2002). In addition, GSK-3 may also regulate PEPCK and G6Pase gene expression in neonatal hepatocytes through the phosphorylation and inactivation of transcription factors, which have potent stimulatory effects on PEPCK gene transcription (Park et al., 1993). The findings of our study revealed that DEX-injected rats showed marked elevation of PEPCK and G6Pase that was dose-dependently reversed by Mang administration. Collectively, glycolysis and glycogen synthesis were induced in the livers of our animals, while gluconeogenesis was blocked by Mang, resulting in the normalization of blood glucose levels, indicating the positive impact of Mang administration in improving IR.

Another molecular mechanism in IR is PPAR-γ/AMPK modulation. AMPK inhibits lipogenesis and enhances fatty acid oxidation to suppress oxidative stress (Gupta et al., 2019). PPAR-γ evokes β-oxidation of fatty acids, increasing glucose uptake and catabolism, and in turn, alleviates non-alcoholic fatty liver disease (NALFD) and improves IR. It has been shown that PPAR-γ regulates the MAPK signaling pathway by inhibiting NF-κB signaling and activating FOXO-1 proteins that result in decreased transcription of IL-1β, leading to critical amelioration of IR-induced inflammatory responses (Soccio et al., 2014). These previous results were supported by our finding that PPAR-γ levels were decreased in hepatic tissues in DEX-injected rats compared to control rats. Improved insulin sensitivity via activating the IRS/AKT/FOXO-1 signaling pathway in aged rat liver resulted in the phosphorylation of FOXO-1 and downregulation of the target gene IL-1β (Padilla et al., 2022). Under conditions of abundant glucose, like during the fed state, insulin is released and promotes activation of AKT in the liver with subsequent FOXO-1 phosphorylation and nuclear exclusion (Cintra et al., 2008; Dong et al., 2008), resulting in inhibition of expression of enzymes responsible for gluconeogenesis (Barthel and Schmoll, 2003). In our study, results indicated reduced hepatic PPAR-γ/AMPK/FOXO1 in DEX-injected rats concomitant with dysregulation in IRS1/AKT, an effect that was reversed upon Mang administration, confirming the effectiveness of Mang in ameliorating IR by modulating the hepatic IRS1/AKT/PPAR-γ/AMPK/FOXO-1 molecular pathway. Our results showed that Mang treatment in DEX-injected rats can effectively combat the impairment of hepatic levels, thus increasing glucose uptake and hence enhancing insulin sensitivity.

Endoplasmic reticulum (ER) stress is known to be increased by obesity and has been proposed to induce IR in the liver and pancreatic β-cells (Craige et al., 2019). ER stress induction suppresses insulin signaling via the serine phosphorylation of IRS1 by JNK, thus interfering with IRS1 function in insulin signaling. ER stress appears to regulate glucose and lipid metabolism, such as lipogenesis, lipid droplet formation, and lipid storage (Lee et al., 2022). Numerous studies have shown a correlation between IR and increasing reactive oxygen species (ROS). DEX-induced hyperglycemia is one of the critical components that extend ROS and lipid peroxidation, causing the exhaustion of the antioxidant defense status in different tissues (Reddy et al., 2009). Our results revealed that the DEX group caused an elevated level of MDA and a decline in antioxidant status, indicated by GSH depletion and reduced SOD and TAC in hepatic tissues compared to control rats. On the other hand, Mang treatment dose dependently restored oxidant/antioxidant balance to near-normal levels. Our findings are consistent with the results of previous studies that found the ability of Mang to increase antioxidant enzymes as well as reduce ROS and MDA in STZ-induced diabetic mice and rats (Pal et al., 2014; Song et al., 2020).

The NLRP3 inflammasome is involved in the regulation of inflammation induced by various non-infectious factors, such as lipid accumulation, oxidative stress associated with hyperglycemia, and hyperlipidemia. The NLRP3 inflammasome facilitates the activation of caspase-1 and secretion of the proinflammatory cytokines IL-1β, IL-18, and TNF-α (Kelley et al., 2019). TNF-α causes hepatocyte necrosis or apoptosis (Shirin et al., 2010), disrupts insulin signaling by promoting serine phosphorylation of insulin receptor substrates, contributing to IR (Plomgaard et al., 2005), and activates the NLRP3 inflammasome, which leads to impaired insulin signaling (Yoon et al., 2022). Our data showed significant elevation in NLRP3/TNF-α levels in the hepatic tissue of the DEX group, promoting inflammation, exacerbating IR, and altering glucose metabolism. Mang administration effectively reduced NLRP3/TNF-α in hepatic tissues. A previous study reported that Mang reduced NLRP3 inflammasome activation in a mouse model of acute hepatic damage induced by lipopolysaccharide and D-galactosamine (Pan et al., 2016) and downregulated NLRP3 gene expression in alcohol-induced hepatitis in rats (Li et al., 2020).

In our study, vascular IR was first confirmed by altered pathological changes in aortic tissues. To further elucidate molecular pathways in this concept, IRS1, AKT, JNK, PPAR-γ, FOXO-1, AMPK, MDA, TAC, SOD, eNOS, NF-κB, TNF-α, ET-1, VCAM, and PGI2 levels were measured. Our data revealed that the previously measured molecular pathway showed reduced aortic levels of IRS1, AKT, PGI2, eNOS, TAC, and SOD and elevated aortic PPAR-γ, FOXO-1, AMPK, MDA, NF-κB, TNF-α, ET-1, VCAM, and JNK levels. In addition, the diminished activity of endothelial progenitor cells (EPCs), derived from bone marrow-derived endothelial stem cells and involved in physiological and pathological angiogenesis (Asahara et al., 1999), was proved by a marked reduction in CD34 and elevation in VEGF to further confirm the mechanism of DEX-induced IR. The previous measured parameter was markedly ameliorated in a dose dependent matter by Mang administration.

The primary risk factors for the generation of endothelial damage are hyperinsulinemia and hyperlipidemia (Chien et al., 1999). According to Laakso et al., elevated insulin concentration can promote VLDL synthesis and hypertriglyceridemia and enhance the formation of LDL-C in the vessel wall. These atherogenic lipid forms, in turn, are involved in the development of lipid plaques in the arterial wall. These fatty changes obstruct the normal blood flow in blood vessels, resulting in cardiovascular abnormalities (Laakso et al., 1991).

The primary explanation for the link between IR and vascular endothelial dysfunction is impaired insulin action due to receptor resistance and its consequent hyperinsulinemia. Under normal conditions, insulin activates downstream signaling through IRS1/PI3K in endothelial cells. Increased JNK activity has been involved in numerous cardiovascular disorders, including IR and endothelial dysfunction (Craige et al., 2019). JNK might serve a dual function as a heterologous inhibitor of insulin action during acute and chronic inflammation and as a feedback inhibitor during insulin stimulation, and these results suggest that activated JNK might be an important negative feedback regulator for insulin signaling. Thus, inhibiting JNK or interfering with JNK-IRS1 interaction might be a good therapeutic target to reduce IR (Lee et al., 2003). In addition, it is reported that the protective effect on cardiovascular disease, especially atherosclerosis, was due to a direct effect of PPAR-γ activation on atheroma and vascular walls but was not a consequence of improving IR. The protection was suggested to be associated with PPAR-γ activation in immune cells and/or vascular cells such as ECs and smooth muscle cells (SMCs) (Stump et al., 2015). In vascular SMCs, the PPAR-γ was reported to directly bind to p65 and facilitate p65 nuclear export rather than the degradation of p65 (Mukohda et al., 2017). As a downstream regulator of the PI3K/AKT pathway, FOXO1 participates in the regulation of the cardiovascular system in a variety of diseases, such as hypertension, cardiac hypertrophy, and atherosclerosis (Li et al., 2014). It is known that the AMPK signaling pathway is associated with vascular smooth muscle contraction and relaxation functions. Vasodilator and hypotensive effects have been reported in various studies using AMPK activators such as AICAR or A769662 (Ford et al., 2012; Schneider et al., 2015). AMPK is proven to be involved in endothelium-independent vasorelaxation in the mouse aorta (Goirand et al., 2007). Vasorelaxation may occur because of mechanisms such as activated potassium channels after the activated AMPK signaling pathway or the eNOS/NO signaling pathway.

One significant pathological mechanism underlying endothelial dysfunction in diabetes mellitus and its related IR states is ER stress (Chen et al., 2022). The relationship between ER stress and insulin-signaling disruption is complex and interdependent. Oxidative stress leads to oxidative alterations in the arterial wall, disrupting intracellular redox homeostasis and causing cellular damage and endothelial cell dysfunction (Taniyama and Griendling, 2003). The pathophysiological mechanisms by which oxidative stress induces endothelial dysfunction include reducing eNOS expression and decreasing NO bioavailability and PGI2 activity. Both are important antiatherogenic factors (Levine et al., 2012). This reduction leads to exaggerated vasoconstriction and induces proinflammatory and thrombotic status (Santillo et al., 2015). In IR, increased vascular tone stemming counteracts NO/PGI2-mediated vasodilation during hyperinsulinemia by impairment of IRS/PI3K/AKT insulin pathway by JNK (Padilla et al., 2022). The downregulation of eNOS expression and activity leads to decreased NO availability along with decreased PGI2 production, contributing to endothelial dysfunction, increased vascular resistance, and heightened susceptibility to cardiovascular diseases (Sena et al., 2013). ET-1 is a potent vasoconstrictor and plays a significant role in regulating vascular tone and blood pressure (Williams et al., 2024). The DEX-induced increase in ET-1 is linked to endothelial dysfunction and may exacerbate hypertension and atherosclerosis (Schiffrin, 2001).

From our data, it is obvious that DEX increased vasoconstrictor factors and reduced vasodilator factors, hence indicating vascular dysfunction. DEX is known to impair endothelial NO production through various mechanisms, including increased oxidation of FFAs in aortic endothelial cells. DEX increases the production of superoxide by the mitochondrial electron transport chain, thus triggering molecular pathways of maladaptive insulin signaling, activating proinflammatory signals, and hampering the activity of key antiatherogenic enzymes such as PGI2 and eNOS. The endothelial AMPK/eNOS pathway accounted for adequate endothelial function in the aorta (García-Prieto et al., 2015). Activation of AMPK in endothelial cells by metformin led to the phosphorylation of eNOS, resulting in the production of NO and subsequent dilation of the mouse aorta (Ewart and Kennedy, 2011). The downregulation of eNOS expression and decreased PGI2 production, along with increased ET-1 level, contribute to endothelial dysfunction.

Another pathophysiological mechanism by which oxidative stress induces endothelial dysfunction is ROS-induced inflammation. The significant elevation of JNK level in the DEX group reflected the proinflammatory and pro-atherogenic environment induced by glucocorticoid treatment (Simoncini et al., 2000). Activation of JNK along with ROS in the aorta is implicated in the development of inflammatory signaling that impairs insulin receptor signaling, contributing to systemic IR (Yang and Trevillyan, 2008; Yung and Giacca, 2020).

NF-κB is an essential member of inflammatory signaling that plays a crucial role in cardiovascular diseases, including atherosclerosis (Broitman et al., 1964; Mallavia et al., 2013; de Souza Basso et al., 2021). Increased levels of TNF-α are associated with impaired endothelial function (Bilsborough et al., 2006). Inflammation contributes to endothelial dysfunction by activating NF-κB, which is translocated to the nucleus and activates its target genes involved in endothelial dysfunction, such as cell adhesion molecules (ICAM-1 and VCAM-1 that facilitate the recruitment of leukocytes to sites of inflammation) and inflammatory mediators like IL-6 and TNF-α. This TNF-α-induced endothelial dysfunction is induced by the production of ROS, and it causes cell death via apoptosis through the activation of NF-κB (Corda et al., 2001). The significant elevation of ET-1 and VCAM levels in the DEX group reflected the proinflammatory and pro-atherogenic environment induced by glucocorticoid treatment. In our results, the heightened levels of aortic NF-κB, TNF-α, ET-1, and VCAM in the DEX group suggested increased vascular inflammation and indicated an increased risk of vascular complications, further exacerbating the negative metabolic effects of glucocorticoids compared to the control group. Thus, it is collectively obvious that DEX-induced IR in rats is associated with cardiovascular disorders in the aorta through modulation of oxidative/inflammatory pathways by elevating the JNK, NF-κB, TNF-α, ET-1, VCAM, and eNOS/PGI2 pathways.

EPCs are circulating cells with the ability to differentiate into mature endothelium and take part in endothelial repair and maintenance. EPCs play an important role in the progression of new blood vessel formation (Lee et al., 2019). A decrease in the number and function of EPCs has been associated with many risk factors for atherosclerosis, diabetes, and insulin resistance syndrome, negatively correlating with homeostasis model assessment of insulin resistance (Loomans et al., 2004; Michowitz et al., 2007). Reendothelialization was significantly reduced in nude nondiabetic mice injected with EPCs from diabetic mice when compared with mice injected with normal EPCs (Zhao et al., 2007; Zhou et al., 2007). EPCs from diabetic patients display functional impairments (Fadini et al., 2006a; Gallagher et al., 2007). Studies looking at the effects of EPC dysfunction in diabetes on endothelial regrowth and neointimal hyperplasia after vessel injury are very few (Boos et al., 2006; Fadini et al., 2006b). However, very little is known about the mechanisms that might lead to the inability of EPCs to induce reendothelialization and decrease neointimal hyperplasia in an inflammatory, insulin-resistant environment. The inability of EPCs to respond adequately to insulin may result in dysfunction in the setting of insulin-resistant states and ultimately lead to increased neointimal hyperplasia postangioplasty. EPCs are characterized by their surface markers CD34 and CD133, as well as by VEGFR2 (Fadini et al., 2008). VEGF is described as an endothelial cell-specific mitogen (Hornig et al., 2000) that stimulates EPC angiogenesis, including survival, motility, and promotes the proliferation of new vessels and increases vascular permeability and is considered to be a critical angiogenic factor of all angiogenesis processes (Shibuya, 2011; Tsai et al., 2020) as well as the transcription factors NF-κB (Tsai et al., 2020). Our results showed that the decreased expression of surface CD34 and increased expression of VEGF as an indication of the inability of EPCs to respond adequately to insulin may result in dysfunction in the setting of insulin-resistant states in DEX-injected rats, which further supports the vascular IR status.

Mang-treated groups improved endothelial function in a dose dependent matter in all previously measured parameters. Previous reports demonstrated the ameliorating effects of Mang on the production of ROS, subsequently altering the MAPK pathway, reducing the activity of eNOS, and reducing antioxidant and anti-inflammatory effects (Bhaskar et al., 2011; Pal et al., 2013; Pal et al., 2014; Yang et al., 2017; Chowdhury et al., 2019; Jia et al., 2019; Sahu et al., 2019).

The significant limitation of this study is that an invasive evaluation of molecular pathways involved in oxidative stress was not performed. Although our data showed that Mang treatment had a potential effect on hepatic and vascular alterations induced by DEX in rats, the impact of Mang treatment on various organ alterations induced by DEX awaits further exploration.

### 5 Conclusion

**FIGURE 15:**
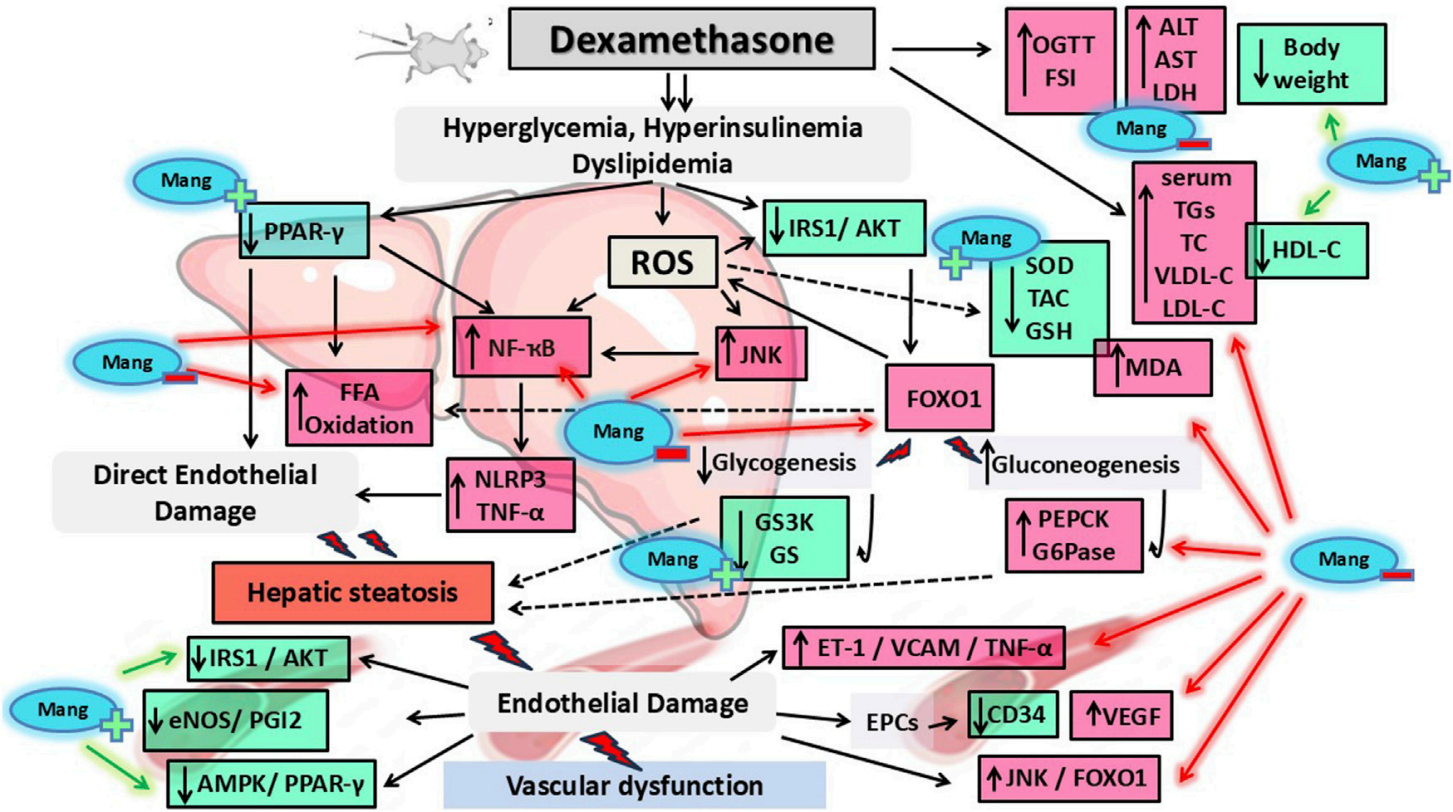
Schematic illustration of the protective mechanism of Mang against DEX-induced IR in rats. AKT: protein kinase B; ALT: alanine aminotransferase; AMPK: AMP-activated protein kinase protein kinase; AST: aspartate aminotransferase; DEX: dexamethasone; eNOS: endothelial nitric oxide synthase; EPCs: endothelial progenitor cells; ET-1: endothelin-1; FFA: free fatty acids; FOXO-1: forkhead box protein O1; FSI: fasting serum insulin; G6Pase: glucose 6-phosphatase; GS: glycogen synthase; GSH: reduced glutathione; GSK-3 glycogen synthase kinase 3; HDL-C: high-density density lipoprotein; IRS1: insulin receptor substrate-1; JNK: C-Jun N-terminal kinases; LDH: lactate dehydrogenase; LDL-C: low-density density lipoprotein; Mang: mangiferin; MDA: malondialdehyde; NF-κB: nuclear factor-kappa B; NLRP3: NOD-like receptor family pyrin domain-containing 3; OGTT: oral glucose tolerance test; PEPCK: phospho-enol pyruvate carboxy kinase; PGI2: prostacyclin; PPAR-γ: peroxisome proliferator-activated receptor-γ; ROS: reactive oxygen species; SOD: superoxide dismutase; TC: total cholesterol; TG: triglycerides; TNF-α: tumor necrosis factor alpha; VCAM: vascular cell adhesion molecule; VEGF: vascular endothelial growth factor; VLDL-C: very-low-density lipoprotein.

In summary, the present study demonstrated that Mang mitigated and reversed IR and associated vascular resistance hepatic steatosis (Figure 15), which were induced by DEX, via mitigating the accumulation of fat and oxidative stress. Mang conferred hepatoprotection via alleviating the associated liver inflammation. This effect might be mediated through inhibition of oxidative stress and NLRP3/NF-κB expression, which suppresses the release of proinflammatory mediator TNF-α, as well as improved insulin-signaling pathway IRS1/AKT and the amelioration of aortic JNK/ET-1/VCAM/eNOS levels, which reflects the anti-inflammatory and antiatherogenic effects. These results provided evidence of the therapeutic potential of Mang in the management of patients with metabolic disorders. More research is required to confirm the protective effect of Mang clinically, pinpoint the exact mechanisms of action, rule out any potentially dangerous side effects for humans, and explore additional molecular pathways.

## Data Availability

The raw data supporting the conclusion of this article will be made available by the authors, without undue reservation.
